# Liquid plasma as a treatment for cutaneous wound healing through regulation of redox metabolism

**DOI:** 10.1038/s41419-023-05610-9

**Published:** 2023-02-13

**Authors:** Hye Ran Lee, Sung Un Kang, Haeng Jun Kim, Eun Jong Ji, Ju Hyun Yun, Sungryeal Kim, Jeon Yeob Jang, Yoo Seob Shin, Chul-Ho Kim

**Affiliations:** 1 Department of Otolaryngology-Head and Neck Surgery, Catholic Kwandong University International St. Mary’s Hospital, Incheon, 22711 Republic of Korea; 2 Department of Medical Sciences, Otolaryngology, Graduate School of Ajou University, Suwon, 16499 Republic of Korea; 3 Department of Otolaryngology, School of Medicine, Ajou University, Suwon, 16499 Republic of Korea; 4 Department of Otolaryngology, College of Medicine, Inha University, Incheon, 22332 Republic of Korea

**Keywords:** Focal adhesion, Integrin signalling, Biomedical materials

## Abstract

The skin functions as the outermost protective barrier to the internal organs and major vessels; thus, delayed regeneration from acute injury could induce serious clinical complications. For rapid recovery of skin wounds, promoting re-epithelialization of the epidermis at the initial stage of injury is essential, wherein epithelial keratinocytes act as leading cells via migration. This study applied plasma technology, which has been known to enable wound healing in the medical field. Through in vitro and in vivo experiments, the study elucidated the effect and molecular mechanism of the liquid plasma (LP) manufactured by our microwave plasma system, which was found to improve the applicability of existing gas-type plasma on skin cell migration for re-epithelialization. LP treatment promoted the cytoskeletal transformation of keratinocytes and migration owing to changes in the expression of integrin-dependent focal adhesion molecules and matrix metalloproteinases (MMPs). This study also identified the role of increased levels of intracellular reactive oxygen species (ROS) as a driving force for cell migration activation, which was regulated by changes in NADPH oxidases and mitochondrial membrane potential. In an in vivo experiment using a murine dorsal full-thickness acute skin wound model, LP treatment helped improve the re-epithelialization rate, reaffirming the activation of the underlying intracellular ROS-dependent integrin-dependent signaling molecules. These findings indicate that LP could be a valuable wound management material that can improve the regeneration potential of the skin via the activation of migration-related molecular signaling within the epithelial cell itself with plasma-driven oxidative eustress.

## Introduction

Skin acts as an outer mechanical barrier against external environmental stimuli, physiologically protecting the body from loss of moisture and electrolytes [1, 2]. In case of acute skin damage typically caused by surgery and trauma, skin wound regeneration prevents secondary infection or damage to internal organs [2]. Rapid skin regeneration leads to recovery from damage as a single organ and contributes to the recovery of impaired body functions and reduction of morbidity and mortality [1, 3]. Given this clinical significance, investigations on the mechanism of skin wound healing and advanced treatment have been conducted.

Acute wound management is aimed at removing inflammatory contaminants and dead tissues by steady wound cleansing and irrigation while providing balanced moisture to create an environment that promotes wound healing [4, 5]. Saline or sterile water has been recommended as a cleansing solution; however, it does not have an additional bioactive effect to improve wound healing [6, 7]. Commercial wound cleansers or antiseptics, such as povidone-iodine, chlorhexidine, alcohol, and hydrogen peroxide, have demonstrated toxicity to normal skin cells, even hindering skin cell regeneration [7, 8]. Host skin cells are central to the re-epithelialization for wound closure. This is done by proper migration and proliferation after the inflammatory phase, including primary hemostasis in the wound healing process [9]. Investigating novel solutions to applied to wound management in clinical practice and promoting self-regenerative potential without harming host skin cells is crucial. Immunomodulatory or antimicrobial agents in the healing process have been developed with an understanding of the underlying molecular mechanisms for acute wound healing and chronicization [10, 11]. However, practically applicable therapy that quickens the healing of acute wounds is currently insufficient. Continuous efforts are required to develop novel treatments that can reduce wound duration in patients.

Plasma is an ionized gas excited by a high energy supply; numerous ions, radicals, and electromagnetic radiation are emitted as effectors along with free electrons [12]. Among them, the redox mechanism induced by reactive oxygen and nitrogen species (RONS) has been suggested as the most important interaction mechanism between plasma and living cells [12, 13]. Plasma has been reported to have an alternative treatment potential for wound healing, sterilization, and anti-cancer purposes; moreover, it is an innovative research field in medicine [12, 14, 15]. Non-thermal gas-type plasma (NTP) has shown a beneficial effect on the highly complex cascade of wound healing [15, 16]. For example, N2-based NTP can facilitate the healing of skin [17] and muscle damage [18, 19]. A study on skin wounds confirmed that the migration of normal skin cells promoted by plasma is important for regeneration [17]. Other studies also reported that NTP treatment promotes cell migration and improves wound healing by changing the activity of molecules involved in cell adhesion and cytoskeletal dynamics of skin cells [20, 21]. However, the method of directly applying gas-type plasma to targets always requires a bulky system, and given that gentle and uniform treatment for a sufficient time is difficult, practical use of this method is limited.

A recent development is the use of liquid plasma (LP), which is also commonly termed plasma-activated water (PAW) or plasma-activated liquid (PAL), generated by gas-type plasma-treatment to liquid media. LP has made the application of plasma easier, and research on whether it exhibits similar activity to gas-type plasma has actively progressed in the biomedicine field [22, 23]. The biochemical activity of LP is an outcome of the physicochemical properties and number of redox molecules generated by the reaction between highly activated plasma gas and water, varying greatly depending on the differences in the LP manufacturing method and process [22, 24]. Regarding the skin wound healing effect of LP, a recent study reported rapid wound recovery by controlling the wound inflammatory response by LP [25]. Another suggested that bacterial inactivation by LP prepared using a dielectric barrier discharge plasma device can support wound healing [26]. Nevertheless, studies on LP activity at the molecular level of host skin cells are insufficient.

Considering this, we aimed to present the improved healing effect on acute skin wounds of LP, which is generated using microwave plasma as a plasma source. Specifically, the migration-promoting effect in skin keratinocytes (HaCaT cells) and the activity of related matrix metalloproteinases (MMPs) and integrin-dependent adhesion-associated molecules were investigated. We analyzed the correlation between their activity and change in intracellular reactive oxygen species (ROS) by determining the redox potential of LP, then examining the regeneration potential of LP via an in vivo experiment using dorsal full-thickness acute skin wound murine models. Overall, we investigated the action mechanism of our novel LP as an effective wound cleansing material that can improve the skin’s regeneration potential.

## Results

### Emission spectrum of the LP system produces RONS

We analyzed various active species generated in our microwave LP system using an optical emission spectroscope (SV 2100, K-MAC, Daejeon, Korea) for emission spectrum analysis. As shown in Supplemental Fig. S1B, the emission spectrum was composed mostly of various molecular and atomic nitrogen and/or oxygen species (NO at 200–300 nm, Second Positive System [N_2_, 300–400 nm], First Negative System [N_2_^+^, 400–500 nm], First Positive System [N_2_, 500–900 nm], O atoms at 777 and 844 nm). Microwave power of 1.2 kW induced the enhancement of molecular oxygen and nitrogen dissociation, represented by a remarkable increase in the emission from the NOγ system at 200–300 nm to a whole range of wavelengths. Nitric oxide derivatives generated by the microwave plasma torch system might subsequently produce RONS in the liquid phase by reaction with water [24, 27]. To detect the ROS generated in LP, we added the Amplex red reagent to the LP and control that changes color after reacting with H_2_O_2_. No color change was observed (Supplemental Fig. S2A). In the nitric oxide assay conducted to confirm the presence of reactive nitrogen species (RNS), only nitrate (NO_3_^−^) was confirmed as an oxidative metabolite of nitric oxide. Nitric oxide-based inhibitors and N-acetylcysteine (NAC) as an ROS inhibitor were treated with LP, and NO_3_^−^ significantly reduced RNS via its inhibitor, MnTBAP [28] (Supplemental Fig. S2B). Thus, among the RONS produced from LP generation, only RNS as NO_3_^−^ could be specified in our experiment.

### LP improves cellular migration potential with increased MMP-2 activities

The skin wound regeneration capacity of LP by activation of keratinocyte migration was confirmed in vitro. We treated HaCaT, an immortalized human keratinocyte cell line, and human skin fibroblasts with LP diluted at 1/2, 1/4, and 1/8 and observed a cell viability similar to that of the control. Similarly, LP did not induce more cell death than the control in the live/dead assay (Supplemental Fig. S3A, B). These results suggested that LP containing active species did not cause a detrimental reaction to the survival of normal skin cells.

**Fig. 1:**
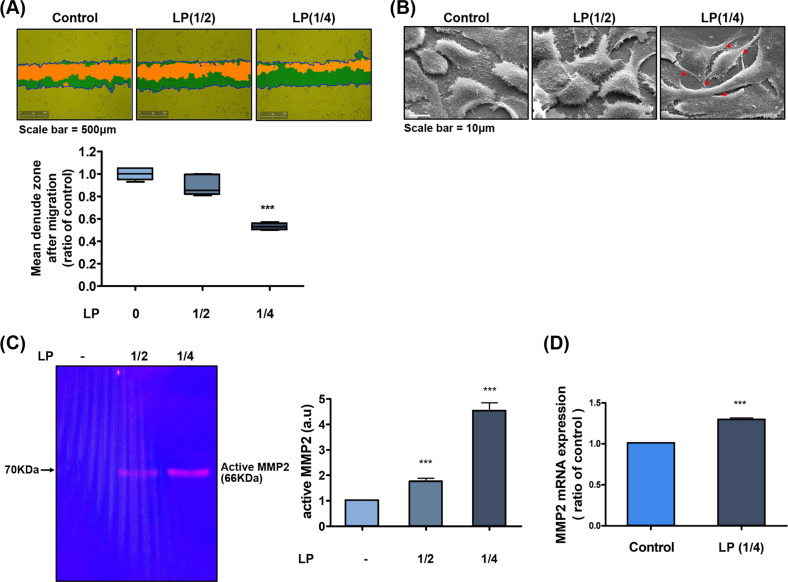
Effects of LP treatment on cell migration. **A** Scratch wound migration assay after LP treatment on HaCaT (human keratinocyte) cells. The cells were plated in six-well plates, grown to 90% confluency, and with a monolayer denuded with a sterile pipette tip. Scratch wound migrations (scale bars = 500 μm) were documented by photography after 24 h of incubation. The data graph presents the mean ± standard deviation of three independent experiments. *P < 0.05, **P < 0.01, and ***P < .001. **B** SEM analysis (scale bars = 10 μm) of morphological changes in cytoskeletal (red indicated) structure in HaCaT cells that were either left untreated or treated with various diluted LP for 24 h. **C** Gelatin zymography for matrix metalloproteinase (MMP) in HaCaT cells. LP increased MMP-2 enzyme activity. Bar graph presents the mean ± standard deviation of three independent experiments. ***P < 0.001. **D** Quantitative PCR analysis was used for the expression level of MMP-2 in HaCaT cells. The mRNA expression of MMP-2 was significantly increased in the group treated with LP. Asterisks indicate statistically significant differences (***P < 0.001).

In the scratch wound-healing assay, as LP-treated HaCaT migration was promoted, the mean denuded zone after migration was reduced more than the control, and the decrease was easily the highest in the four-fold diluted LP (Fig. 1A). In addition, migration was increased in primary keratinocytes and primary fibroblasts compared to the control group when treated with 1/4 diluted LP (Supplemental Fig. S4A, B). When comparing the two cells, since HaCaT cells were more active in promoting cell migration by LP than primary keratinocytes, we decided on HaCaT cells as the subject of all subsequent experiments. The pattern of keratinocyte migration was confirmed in real-time for 24 h via interval time-lapse microscopy, and a faster in-growth rate of scratch wounds was observed in the LP-treated group (Supplemental Fig. S5). In keratinocytes treated with various concentrations of LP, there were no significant changes in cellular proliferation as another mechanism was involved in the wound-healing process, and expression of proliferating cell nuclear antigen (PCNA) (Supplemental Fig. S6). In the transmission electron microscopy imaging of wound-edge keratinocytes showing migration activity, the LP-treated group showed a marked increase in the intercell contact area and the conversion to an elongated morphology for spreading. Furthermore, we observed lamellipodia and filopodia, which are protruded cellular membrane structures generated by actin turnover for migration [29]. These changes were most pronounced in the 1/4 diluted LP (Fig. 1B). MMPs of the extracellular matrix (ECM) were involved in the tissue remodeling for wound healing, and we observed changes in the expression of MMP-2, known to promote keratinocyte migration as gelatin-binding MMPs, via gelatin zymography and RT-PCR [29, 30]. As shown in Fig. 1C, MMP-2 activity in zymography was significantly increased in LP-treated groups than that in the control group. In the RT-PCR analysis of the 1/4-diluted LP-treated group, which showed increased MMP-2 activity than that of the 1/2-diluted LP; the MMP-2 mRNA expression was significantly higher than that of the control (Fig. 1D). These results suggested that the enhanced enzymatic activity and transcription of MMP-2 contributed to the acceleration of keratinocyte migration by LP. We performed subsequent experiments with 1/4-diluted LP, which led to the most remarkable findings in the scratch wound-healing assay while evaluating MMP-2 activity.

### LP activates integrin-dependent cell spreading and migration

**Fig. 2:**
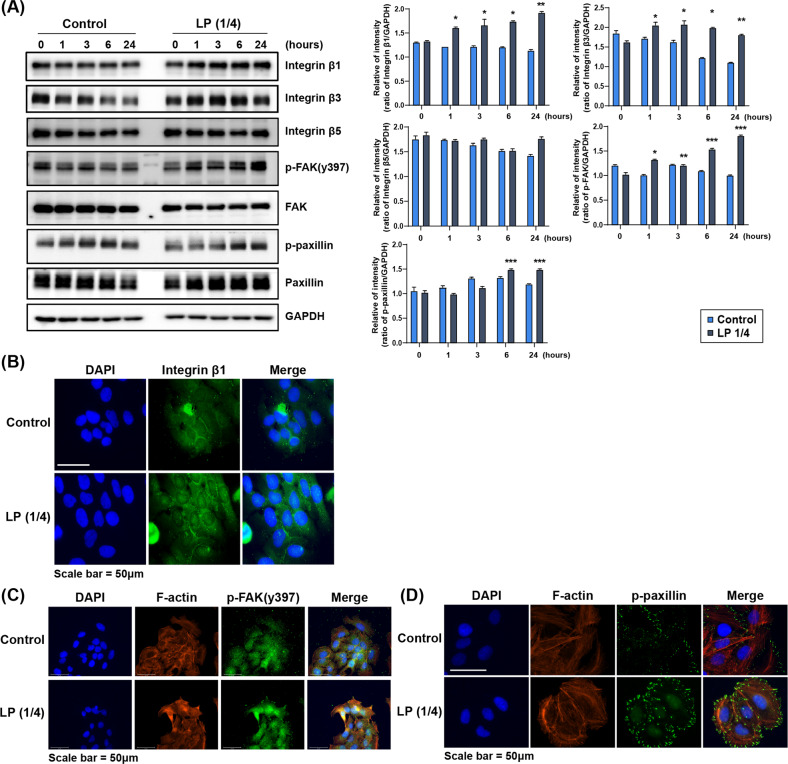
LP increases the expression of proteins involved in extracellular matrix (ECM) signaling. **A** Expression of proteins involved in ECM signaling in HaCaT cells was confirmed by western blot. The degree of protein expression of integrin β1, p-FAK, and p-paxillin was significantly increased with LP (1/4) treatment time than the control group. All the western blotting experiments were performed under the same condition. After transferring the blots onto PVDF membranes, we immediately cropped the targeted blots according to referenced indicating markers. Targeted proteins were immunoblotted with the specific antibody for protein normalization. **B** Integrin β1 protein levels were determined by immunocytochemistry after LP (1/4) treatment for 24 h. Representative fluorescence microscopic images of HaCaT cells are shown. Scale bars = 50 μm. The increase in the expression of ECM molecule after LP treatment was confirmed using immunofluorescence assay. The green-stained stock in the cytoplasm represents p-FAK (**C**), and p-paxillin (**D**). The amount of green-stained stock increased in the LP-treated group. We performed Texas-Red conjugated phalloidin to visualize the cytoskeleton (F-actin) and used Hoechst 33258 to label cell nuclei. Each figure is representative of three experiments with triplicates.

Integrin beta(β)-subunits, especially β1, FAK, and paxillin, are molecules that constitute the focal adhesion complex on the keratinocyte surface and are involved in the cascade of cell migration [31, 32]. A previous study found that N_2_-based NTP inhibits the migration of oral squamous carcinoma cells, simultaneously decreasing the expression of integrin β3, phospho(p)-FAK, and phospho(p)-paxillin [33]. In this study, for further investigation into the molecular mechanism by which LP promotes keratinocyte migration, we confirmed the expression changes of integrin β-subunits, p-FAK, and p-paxillin in LP-treated keratinocytes by western blot and immunofluorescence analysis. The expression change of each molecule was observed during LP treatment for 24 h. In the control group, we found no difference in the amount of expression change with time and noted a decrease in expression level, such as in integrin β3. In the LP-treated group, the expression of integrin β1, β3, p-FAK, and p-paxillin increased over time (Fig. 2A). Alterations in the cytoskeletal structure of LP-treated migrating keratinocytes were visualized by F-actin immunofluorescent staining. We also investigated the co-expression patterns of integrin β1, p-FAK, and p-paxillin. As shown in Fig. 2B, notably increased integrin β1 expression could be observed throughout the cytoplasm in the LP-treated group compared with the control group. Owing to the growth of small F-actin filament bundles, filopodia were actively formed (Fig. 2C, D) [34], and the manifestation of p-FAK and p-paxillin was concentrated on the cytoplasmic surface along the contour of the re-organized actin cytoskeleton. Taken together, LP treatment stimulated cytoplasmic focal adhesion molecules, inducing the signaling pathway in the direction of promoting keratinocyte migration and in concert with actin filament-based cytoskeletal reassembly.

### Accelerated cell migration by LP via intracellular ROS signaling

**Fig. 3:**
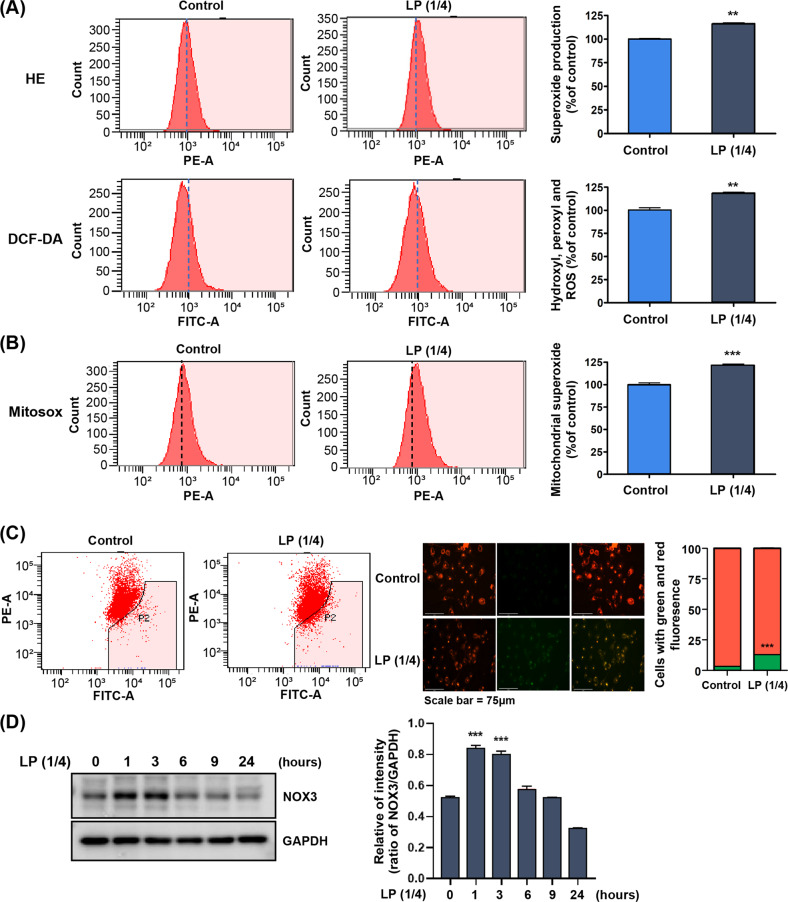
Treatment of LP increases cellular oxidative stress and mitochondrial membrane potential (∆Ψm). **A** The HaCaT cells treated with LP for 24 h were analyzed using flow cytometry, with dihydroethidium and 5-(and-6)-carboxy-2’,7’-dichlorodihydrofluorescein diacetate staining. **B** For the measurement of mitochondrial superoxide, the cells were incubated with 2.5 mM of MitoSOX and then stained and analyzed using flow cytometry. In (**A**) and (**B**), the data graph presents the mean ± standard deviation of three independent experiments. ***P < 0.001. **C** Measurement of ∆Ψm with JC-1. The JC-1 fluorescence shifted from red-orange to green, indicating depolarization of the ∆Ψm. The ∆Ψm change was measured objectively using a fluorescence microscope and flow cytometry. Bar graph presents the mean ± standard deviation of three independent experiments, calculated as a percentage of the control. **P < 0.01, ***P < 0.001. **D** Intracellular ROS stimulated with LP induces expression of NOX3 protein in HaCaT cells. Western blotting analysis for NOX3 antibody. GAPDH was used as the loading control.

Intracellular ROS activation has been a suggested mechanism by which plasma application regulates cell activities. Active research has attempted to elucidate the cellular redox signaling by intracellular ROS [35, 36]. Intracellular ROS functions as a mediator of cellular adhesion and migration by integrins and their downstream pathways [37, 38]. Consequently, we quantified intracellular ROS expression of LP-treated keratinocytes by HE and DCF-DA staining and observed significant increases of superoxide and other ROS, including hydroxyl and peroxyl, within the cell in the LP-treated group compared to the control (Fig. 3A). In mammalian cells, mitochondria are a key source of ROS, and their activity is regulated in response to adenosine triphosphate, acting as a regulator of the intracellular ROS level [39, 40]. We quantified the superoxide, a mitochondrial ROS (mtROS), in the LP-treated cells by MitoSOX red mitochondrial superoxide indicator. As shown in Fig. 3B, the measured amount significantly increased compared with to the control.

The generation of excessively high intracellular ROS is related to various pathologies associated with oxidative stress [41]. We investigated the alteration of the mitochondrial membrane potential (∆Ψm) as the regulatory activity of redox homeostasis. The ∆Ψm-sensitive probe, JC-1, forms red fluorescent J-aggregates at high membrane potential, whereas at lower potential, it is detected as a green-fluorescent monomer. In our experiment, LP-treated keratinocytes showed a higher green/red fluorescent ratio by JC-1 staining than the control group, which demonstrated reduced ∆Ψm (Fig. 3C). This result suggested the alteration and activated the regulatory process of mitochondrial redox status by LP. We then verified the activity of NADPH oxidase (NOX), which are enzymes participating in intracellular redox homeostasis regulation besides mitochondria and are well-known for responding to various cellular stress stimuli [42, 43]. We observed the expression pattern of NOX3, a homolog of the NOX family, in LP-treated cells; these cells showed that NOX3 expression in western blot was markedly increased up to 3 h immediately after treatment and decreased subsequently (Fig. 3D). Taken together, these results indicate that LP treatment promoted intracellular ROS generation in keratinocytes, where the mitochondria and NOX family were involved together and concurrently played a role in maintaining redox homeostasis.

### ROS scavenger incapacitates the keratinocyte migration activated by LP

**Fig. 4:**
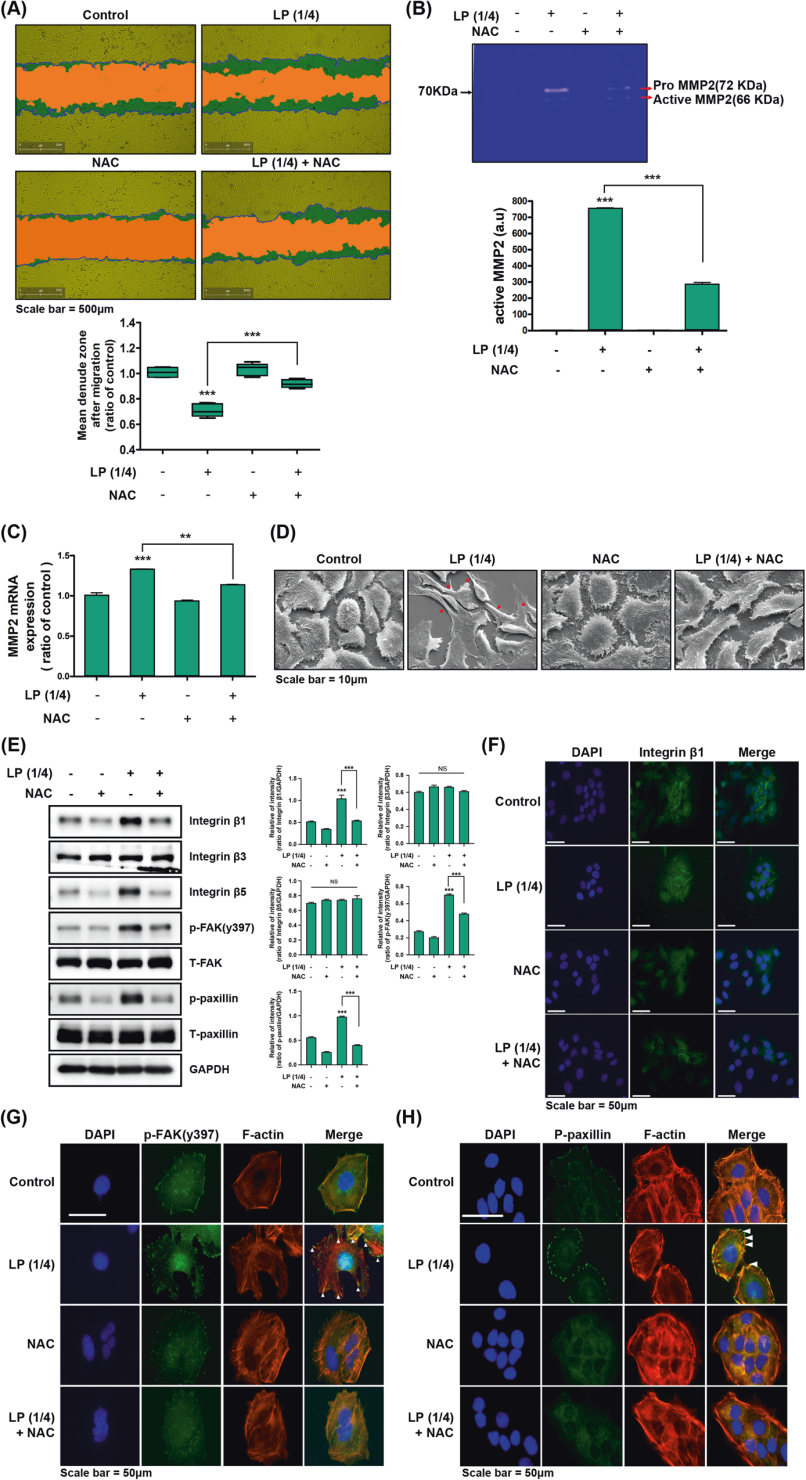
Correlation among LP-induced cellular ROS generation and ECM signaling activation. The HaCaT cells were incubated for 1 h before treatment with LP in the presence or absence of NAC (10 mM). **A** Scratch wound migration assay after LP treatment on HaCaT (human keratinocyte) cells. LP significantly increased cell migration across the cell stripped area, but significantly attenuated it when treated with the ROS scavenger, NAC. **B** Gelatin zymography for MMP-2. NAC attenuated the enzymatic activity of MMP-2 increased by LP. Bar graphs represent the mean ± standard deviation of three independent experiments. ***P < 0.001. **C** Quantitative PCR for MMP-2. The mRNA levels of MMP-2 decreased after NAC treatment. Each figure is representative of three experiments with triplicates. **P < 0.01, ***P < 0.001. **D**, **B** SEM images confirmed the effect of NTP on cell morphology. The cytoplasm of LP-treated HaCaT cells showed an increase in horizontal polarization and contraction and cytoplasmic protrusion compared with the control group (red arrow), but it was significantly inhibited in NAC-treated cells. **E** Western blot analysis. Integrin β1,3,5, p-FAK, and p-paxillin expression were suppressed significantly after NAC treatment to HaCaT cells. **F**–**H** Immunofluorescence analysis confirmed whether NAC could attenuate the expression of integrin β1 (**F**) and ECM molecules (p-FAK (**G**), and p-paxillin (**H**)) increased by LP. We performed Texas-Red conjugated phalloidin to visualize the cytoskeleton (F-actin); we used Hoechst 33258 to label cell nuclei. Each figure is representative of three experiments with triplicates. Scale bars = 50 μm.

We conducted the loss of function test using N-acetylcysteine (NAC), a well-known ROS scavenger, to reversely confirm whether cell migration signaling was triggered by intracellular ROS, whose expression was increased by LP inducing ROS deprivation. Figure 4A shows the results of the scratch wound healing assay performed with NAC, in which keratinocyte migration was significantly reduced when LP and NAC were treated together, compared to the condition where cell migration was promoted by LP treatment alone. Notably, the NAC-only treated cells showed little difference in the mean denuded zone area after migration compared to the control, thereby proving that the migration-promoting effect of LP was suppressed by NAC. Active-MMP-2 expression in gelatin zymography decreased in the LP and NAC co-treated group, representing a result opposite to that of the LP-only treated group, which showed a higher expression (Fig. 4B). In the RT-PCR, MMP-2 mRNA expression showed the equivalent pattern as the zymography and was significantly lower than the LP-only treated group by NAC co-treatment (Fig. 4C). We found no significant change in the MMP-2 expression amount in the control and NAC-only treated groups in both experiments. In the SEM imaging of migrating keratinocyte co-treated with LP and NAC, and NAC-only treated cells, the LP-induced morphological change to an elongated shape and development of lamellipodia and filopodia were hardly observable (Fig. 4D). The expression levels of integrin β1, p-FAK, and p-paxillin—molecules that were identified to be increased by LP in the previous western blot—decreased to the level of the control or NAC-alone treatment group when NAC treatment was added with LP (Fig. 4E). We then confirmed these findings by performing an immunofluorescence assay. Consistently, Fig. 4F–H show the expressions of integrin β1, p-FAK, and p-paxillin visualized by immunofluorescent staining. The overall increase in integrin β1 expression in cytosol with LP treatment was notably reduced when NAC and LP were treated together, and cells with no expression were also found (Fig. 4F). p-FAK and p-paxillin immunofluorescence, which characteristically appeared at a prominent expression level in the cytoplasm along the F-actin structure following LP treatment, were also lost after NAC treatment (Fig. 4G, H). Therefore, in the above experiments, as simultaneous treatment of LP and NAC inhibited the integrin β1-dependent intracellular signaling associated with increased keratinocyte migration by LP, we determined that intracellular ROS mediated keratinocyte migration signaling.

### LP enhances acute wound regeneration of skin cells in the murine dorsum

**Fig. 5:**
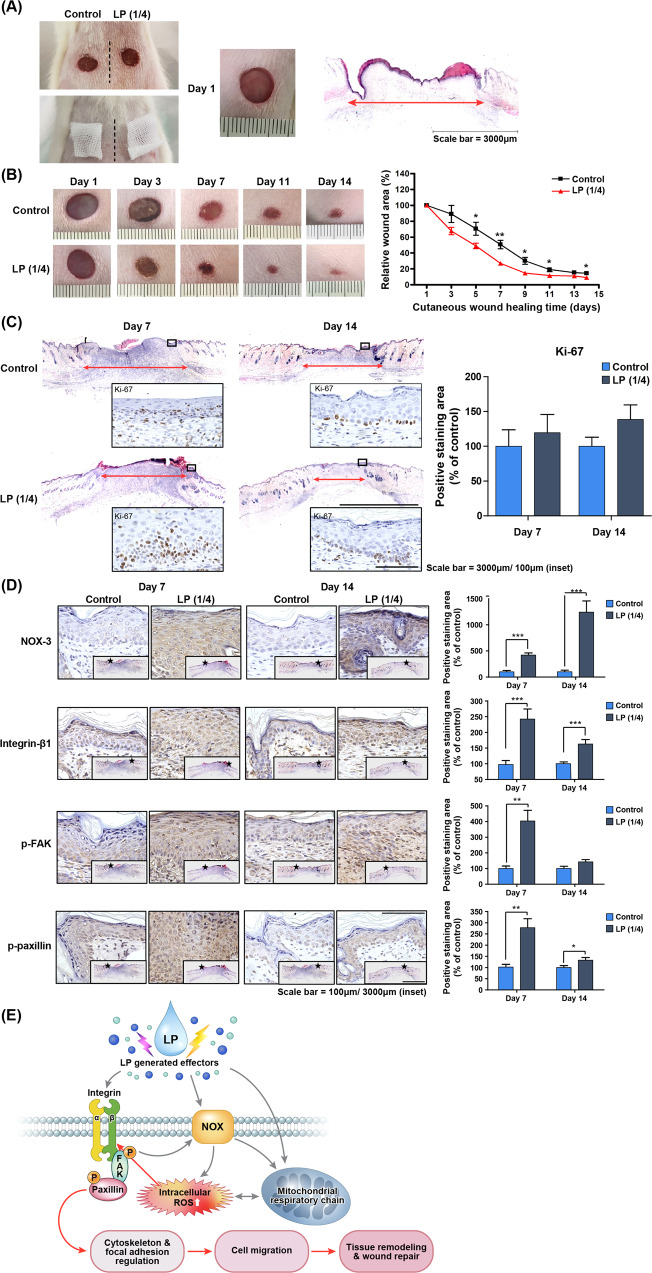
LP treatment promoted wound regeneration of murine dorsal acute skin wounds by activating intracellular ROS and epithelial cell adhesion molecules. Twenty rats were separated into untreated control (*n* = 10) or LP treatment (*n* = 10) groups. **A** Images represent control and LP-treated wounds in rat dorsum. Two full-thickness wounds, 8 mm in diameter, were created on both sides of a rat’s upper back. Left: photographs of both sides of control and LP-treated (upper) and PBS (control) or LP-soaked gauze applied wounds (lower). Right: photograph of initially created wound on day 1 and its cross-section histologic image. Scale bar = 3000 μm. **B** Photographs of dorsal skin wound closures (left), which were controlled and treated with LP on days 1, 3, 7, 11, and 14. Quantification of the relative wound area (%) to the initial wound in control and LP-treated wounds (right). Mean ± SEM, *P < 0.05, **P < 0.01. **C** Left: On days 7 and 14 after wound creation, the wounds’ cross-sections stained by H&E staining showed decreased wound diameter in the LP-treated group. Ki-67 expressions were detected by IHC in each wound epithelium (black squares) and demonstrated increased expression in the basal layer of the epithelium in the LP-treated group. Scale bar = 3000 μm (H&E images)/100 μm (IHC images). Right: Positive immunostaining area of Ki-67 in both control and LP-treated groups on days 7 and 14 represented graphically. Mean ± SEM. **D** Left: To evaluate the effect of LP treatment on wound tissues, we performed NOX3, integrin β1, p-FAK, and p-paxillin IHC staining in both control and LP-treated groups on days 7 and 14. The IHC images in each box are 10× the magnification of the star-marked area in inserted H&E images of each box with an original magnification of 40×. Scale bar = 100 μm (IHC images), 3000 μm (H&E images). Right: Positive immunostaining area of each marker in both control and LP-treated groups on days 7 and 14 represented graphically. Mean ± SEM, *P < 0.05, **P < 0.01, and ***P < 0.001. **E** Schematic of LP treatment on keratinocytes and intracellular ROS mediated adhesion molecule activation as a subsequent process for promoting cell migration and wound regeneration.

As shown in Fig. 5A, round, full-thickness skin wounds with a diameter of 8 mm were created on both sides of the dorsum of the rat. Gauze soaked in LP was applied to the right wound for 15 min every two days, and the wound healing process was observed for 14 days. The left wound was then treated with PBS in the same way as the control. The wound area closed more rapidly from the start of wound healing in the LP-treated group, and a significant decrease in the wound area was confirmed from days 5 to 14 of observation compared to the control group. The wound depth became shallower along with accelerated skin regeneration; the scar visible on day 14 was milder in the LP-treated group (Fig. 5B). The tissue was obtained on days 7 and 14 of wound creation. Histologic analysis was performed with hematoxylin and eosin staining. On both days 7 and 14, the length of the wound section on the tissue was more reduced in the LP-treated group than in the control group (Fig. 5C). Immunohistochemistry analysis was performed with Ki-67 cell proliferation marker, and the expression of Ki-67 in the basal layer of the epithelium was increased in the LP-treated group on both days 7 and 14 compared to the control group. On day 7, a thicker keratinocyte epithelial layer was formed, and Ki-67 expression was active, suggesting that the proliferation of healed wound epithelium was active. On day 14, after continuous LP treatment, the expression level of Ki-67 and the total thickness of the epithelial layer decreased, but the maturation of the epithelial layer and transition to the keratin layer were occurring in a stable manner. Particularly, the proliferation potency of the epithelial basal layer for further regeneration was maintained on day 14 in the LP-treated group (Fig. 5C). The changes in the expression profiles of NOX3 and integrin β1 with sub-signal molecules p-FAK and p-paxillin in the wound epithelium are shown in Fig. 5D, prepared by immunohistochemistry staining and quantification of the results. In both tissues on days 7 and 14 from wound creation, each molecule increased more in the LP-treated group than in the control, and all increases were statistically significant, especially on day 7. On day 14, the increment in the expression of all molecules in the LP-treated group was statistically significant, except p-FAK. Figure 5E schematically delineates the results of the in vitro and in vivo experiments, in which wound regeneration is accelerated with keratinocyte migration promoted by LP treatment and the mechanism thereof. Treatment with LP activated intracellular ROS-generating systems, including the NOX family of cell membranes and mitochondria. Activation of the β-subunit of integrin by increased intracellular ROS led to phosphorylation of the sub-signal molecules in the integrin adhesion complex, FAK, and paxillin, thereby causing cytoskeletal morphogenic change and focal adhesion regulation in a direction that promoted cell migration. These mechanisms collectively contributed to rapid tissue remodeling and wound repair.

## Discussion

Keratinocytes are the most dominant cells in the epidermal layer of the skin. They play a crucial role in the re-epithelialization for wound closure during skin regeneration of wounds [44]. A core mechanism of re-epithelialization which is the epithelial-mesenchymal transition—the migration of wound-edge keratinocytes into the wound bed [44, 45]. The denuded wound area is primarily covered by keratinocyte migration and subsequent proliferation and is followed by maturation of the wound through a remodeling phase that includes matrix formation [46, 47]. Intricate interactions between the cellular components of the skin, the ECM to regulate their activity, and the associated molecular mechanisms are simultaneously involved in these tissue repair processes [46, 48]. Given that the re-epithelialization helps in the functional recovery as maintenance of the epithelial barrier function, the activation of molecular signaling factors in the direction of steering keratinocyte migration, and appropriate induction of cytoskeletal reformation of the cell itself, comprise an important strategy for improved wound healing [34, 49, 50]. We confirmed that cellular migration was promoted by LP treatment on HaCaT cells. The cells were transformed into structures favorable for migratory activity, including the development of cytoplasmic protruding structures. Therefore, we attempted to verify the intracellular migration-promoting molecular dynamics in keratinocytes activated by LP treatment during the regeneration of cells in acute skin wounds.

The migration of wound-edge keratinocytes is preceded by the release of cell–matrix adhesion by the wounding of the epidermis, which is modulated by multiple regulators [46, 49]. Regulators mediating intercellular interactions with immune cells recruited by acute skin injury include growth factors, cytokines, and chemokines, which activate keratinocyte transformation to the migration-prone phenotype [50, 51]. Integrins, FAK, and paxillin—as core adaptor cytoplasmic proteins that constitute the focal adhesion complex—are involved in the modulation of actin cytoskeletal organization, attachments to the ECM, and in signaling pathways that control the migration direction and extent of epithelial cells in wound healing [2, 31, 52, 53]. Integrins are heterodimeric transmembrane glycoproteins composed of α and β sub-units that act as intermediate conduits for bidirectional signal transduction between the inside and outside of cell membranes [52]. The overlapping of multiple combinations of 18 α and 8 β sub-units acts on critical processes in epidermal wound healing, such as cellular migration, proliferation, and matrix remodeling [52, 54]. The importance of integrin β1 in wound re-epithelialization has been consistently demonstrated. Studies using the integrin β1 gene-knockout murine model have indicated a decrease in keratinocyte migration and cytoskeletal re-orientation and impairment of skin organization attributable to basal keratinocyte dysfunction [55, 56]. Furthermore, they have established the mechanisms of the activation of keratinocyte migration by phosphorylation of FAK and paxillin, the components of the focal adhesion complex incorporated in the downstream signaling cascade of integrin β1, and the accompanying reassembly of actin fibers promoting cell–ECM interaction and cytoskeletal reorganization [52, 53, 57, 58]. We observed in both in vitro and in vivo experiments that the cellular adhesion-regulating molecules integrin β1, p-FAK, and paxillin increased, while keratinocyte migration was promoted by LP treatment. The manifestation of lamellipodia and filopodia by additional actin reorganization could also be visualized. The more pronounced expression of migratory cascade molecules and proliferation markers in keratinocytes on day seven than on day 14 in the in vivo IHC reflects the progression of a new tissue formation stage by the re-epithelialization from days 2 to 10 of wound creation [46].

Direct treatment of gas plasma generated by ionization of existing non-thermal atmospheric pressure gas promotes migration and proliferation by regulating the adhesion-dependent signaling of keratinocytes and fibroblasts participating in skin wound healing [53, 59–61]. As a mechanism, regulation of the ERK/MAPK pathway or epithelial-mesenchymal transition and cell cycle has also been suggested, in addition to integrin-dependent signaling [60, 61]. Meanwhile, studies have confirmed that additional beneficial effects of plasma treatment can be expected on regenerative microenvironments, such as anti-inflammatory effect and neo-angiogenesis [59, 62]. Scholars continue to investigate the efficacy of plasma-activated solution with improved applicability and storability to living tissue, which can be used for indirect treatment through delivery in combination with other materials [22, 24, 63]. The higher permeability through the epidermis compared to the direct treatment of conventional gas type plasma can be another advantage of applying LP to skin wounds [64–66].

Our study showed a significant improvement in murine dorsal wound skin cell regeneration and maturation rate through LP treatment. We demonstrated the activation of molecules involving integrin-dependent migration cascades in wound tissue and confirmed the mechanism of our solutionized plasma in promoting wound healing by controlling the activity of host epithelial cells at the molecular level. Moreover, we demonstrated that LP has potential as an innovative plasma material that could reproduce, in a stable manner, the effect of cellular activation by gas-type plasma established in previous studies. Several studies have reported that bacterial deactivation of plasma can attenuate wound inflammation and promote tissue repair as a result [26, 67]. LP also has a wide range of bactericidal effects while protecting normal skin cells [68]. Therefore, apart from the activated migration cascade of keratinocytes, the additional effect of suppressing tissue repair obstacles, such as bacterial contamination and inflammation, may have a positive effect on improving wound healing.

Studies have reported on the mechanism by which plasma interacts with living cells to generate a biologic-cellular response that promotes skin regeneration. Among the effectors of plasma, the role of ROS, an essential intracellular moderator of cellular metabolism, has been suggested representatively [53, 69]. ROS orchestrates the biophysiological activity of immune and non-immune cells involved in the normal wound healing process at the wound edge [62, 70, 71]. ROS comprehensively regulates the formation of cellular protruding podial structures by rearranging the actin cytoskeleton via the downstream action of subsequent signal molecules involved in cellular migration. This includes the integrins cluster and FAK of the focal adhesion complex [37, 38, 72]. Our experiment also confirmed that integrin-dependent cascades were inhibited by NAC, a ROS scavenger treated with LP, besides the direct increase of intracellular ROS by LP. This could prove that the activation of integrin-dependent cell adhesion molecules is a ROS-dependent cellular response induced by LP.

By external stimuli, including plasma, intracellular ROS is generated by cell organelles, such as mitochondria and peroxisomes, and specialized enzymes including NOX [71, 72]. NOX is stimulated by cellular stress environment, as in skin wounding, and ROS produced by NOX is actively involved in the regeneration of injured tissue by modulating intracellular redox-dependent signaling cascades [35, 42]. This controlled stimulus of physiological low-level intracellular ROS—the maintenance of oxidative eustress—promotes cellular differentiation and tissue regeneration, whereas oxidative distress caused by excessive ROS induces pathogenesis by cellular dysfunction and eventual cytotoxicity [62, 73]. Thus, the regulatory mechanisms of ROS and redox responses must be maintained at homeostatic levels to maintain the normal functioning of cells [39, 74]. In the “ROS-induced ROS release” process of mitochondria, activated ROS burst, caused by stimuli like plasma, opens the mitochondrial permeability transition pores, which temporarily increases mtROS and simultaneously decreases ∆Ψm [39, 75]. This mechanism serves as a buffer to prevent irreversible injury from ROS accumulation in the mitochondria followed by ROS accumulation [39]. Likewise, endogenous antioxidants for maintaining each physiologic ROS level according to cell type prevent oxidative distress [71, 74]. In our experiment, we observed that NOX activation and intracellular ROS increased within several hours after LP treatment was followed by redox homeostasis regulation, represented by an increase in mtROS and a decrease in ∆Ψm.

To investigate the key effector of LP outside the cell, which induces intracellular ROS activation and subsequent cellular cascade, we conducted nitric oxide assay for investigating RNS, a known representative effector of plasma along with ROS [22]. Among the reactive species emitting from LP, only RNS as NO_3_^−^ could be identified in our experiment. A previous study confirmed that NO_3_^-^ isomer in LP exhibits antibacterial activity but does not inhibit the viability of normal skin cells [68]. Other studies have demonstrated that RNS, especially NO, promotes wound healing. A recent study reported that stable RNS-enriched, plasma-activated liquid media can promote astrocyte wound healing by cellular migration and proliferation cascade [76]. RNS represented by NO_3_^−^ may be an effector mediating the effect of LP to encourage wound regeneration, supporting the role of RNS suggested in former studies. However, the linking mechanism by which extracellular RNS delivered by LP promotes intracellular ROS-dependent cellular growth will require further investigation.

Taken together, our study suggested that LP is an innovative material that can contribute to rapid skin cell regeneration in acute wounds by effectively promoting re-epithelialization without epidermal cell damage, while inducing oxidative eustress at a physiological level. Delayed wound healing is a serious clinical problem, as it initiates a chain of events that culminate into fatal complications from vital organ exposure, especially in patients with reduced resilience to wounds, such as aged and diabetic populations. Recently, randomized clinical trials of plasma therapy for wound healing in diabetic foot ulcers have been conducted [77], and there are continuous attempts to apply plasma to clinical wounds. To extend the scope of application of LP in actual clinical practice, researchers should seek an in-depth understanding of the mechanism of action and effectors of LP. In addition, it is essential to set an appropriate concentration of LP that can maximize the desired effect without damaging normal tissue. Hence, the experimental role before clinical application is emphasized in this regard.

## Materials and methods

### Design of liquid plasma (LP) and treatment

We designed a microwave LP system as previously described to advance research for biological research applications [22, 78]. Briefly, it has a frequency of 2.45 GHz, a micropower of 1.2 kW, and air is injected at 20-LPM (liters per minute) per minute. Thereafter, the gas generated from the plasma torch flame at a temperature of 6000 K flows through the cooler into the LP tank to generate plasma water (Supplemental Fig. S1A). LP was treated by dilution to various concentrations using 2× concentrated medium and incubated for an additional 24 h. As a relevant control, studies were performed using 2× concentrated medium alone under the same experimental conditions.

### Cell culture

The HaCaT human keratinocyte cell line was obtained from the Korean Cell Line Bank (Seoul, Korea). The cells were grown in Dulbecco’s modified Eagle’s medium with 10% fetal bovine serum and 100 U/mL penicillin-streptomycin at (Gibco, Paisley, PA, USA).

The Human Primary Dermal Fibroblast Neonatal (HDFn, PCS-201-010) cells and Primary Epidermal Keratinocytes: Normal, Human, Adult (HEKa, PCS-200-011™) were obtained from the ATCC (Manassas, VA, USA) as a comparative cell. The Fibroblast cells were grown in Fibroblast Growth Kit–Serum-Free (ATCC® PCS-201-040), and Keratinocyte cells were grown in EpiLife Medium supplemented with human keratinocyte growth supplement (Gibco, Paisley, PA, USA) at 37 °C in a humidified atmosphere with 5% CO_2_/95% air.

### Cell viability

Cell viability was tested using MTT solution (3-(4,5-dimethylthiazol-2-yl)-2,5-diphenyl-tetrazolium bromide) (Sigma, St. Louis, MO). Cell viability assays were performed as described previously [79]. Optical density (OD) was measured for each culture well, using a microplate reader (Bio-Tek, Winooski, VT, USA) at 540 nm. Cell Viability analysis was performed using the Muse™ Cell analyzer (Millipore, Massachusetts, USA) following the manufacturer’s protocol.

### Cell migration assay

Cell migration assays were performed as described previously [80]. Briefly, 1 × 10^5^ cells were plated on 6-well plates and grown to 90% confluency for 2 days followed by serum deprivation for 1 h. Wounds were generated using a sterile 1000 ml pipette tip and washed with growth medium, and cells were treated with diluted LP to 1/2, 1/4, respectively. Three wells per experimental treatment were examined at ×10 magnification by light microscopy (EVOS FL auto, Thermo Fisher). Each experiment was performed in triplicate.

### Gelatin zymogram assay

MMP2/9 activities were assayed using gelatin zymogram assay, as described previously [80]. HaCaT cells were treated with diluted LP to 1/2, 1/4, incubated for an additional 24 h. The supernatant (100 μL) from each sample was mixed with 4-aminophenylmercuric acetate (100 μM, Sigma-Aldrich, St Louis, MO, USA), and the samples were activated for 1 h at 37 °C. The sample was placed in sample buffer for 10 min and electrophoresed on a polyacrylamide gel at 125 V for 120 min at 4 °C using a Novex XCell II system (Life Technologies, Carlsbad, CA, USA). Digital images were taken using an image analyzer.

### quantitative RT-PCR (real time-PCR)

Total RNA was extracted from human keratinocyte cells treated LP using Trizol reagent (Thermo Fisher Scientific). Real time PCR were performed as described previously [79]. We quantified the targeted genes with one-step realtime PCR using a Lightcycler 96 (Roche Molecular Biochemicals, Basel, Switzerland). PCR primers were purchased from Qiagen (Qiagen, Germantown, MD, USA); GAPDH mRNA levels were used for normalization.

### Western blot

Western blot was performed as described previously [80]. The antibodies we used are listed in Supplementary Table 1. Exposure times and amounts of protein loaded were optimized so that optical densities were linear, as determined by performing a dilution of a representative antibody and protein sample.

### Immunocytochemistry

After culturing on a microscope coverslip (Thermo Fisher Scientific, Rochester, NY, USA) and treatment of diluted LP (1/2, 1/4). After 24 h, the coverslips were washed with 1× PBS, fixed for 1 h in 4% paraformaldehyde. After blocking for 45 min in BSA (in 5% PBS), the slides were incubated for 2 h with anti-Integrin β1, p-FAK and-paxillin antibodies (1:50; Cell Signaling Technology), washed with 1× PBS, and incubated for 45 min with secondary antibodies (1:250; Invitrogen). After rinsing in PBS, Hoechst 33258 was added for 15 min to counterstain the nuclei. The slides were washed with PBS and mounted with Vectashield (Vector Laboratories, Inc., Burlingame, CA, USA, H-1000) then analyzed using fluorescence microscopy (EVOS FL auto, Thermo Fisher).

### Measurement of intracellular ROS production

The cellular ROS production was measured by treating HaCaT cells with 10 μM hydroethidine (HE; Molecular Probes, Eugene, OR) for 30 min at 37 °C. Fluorescence-stained cells were then analyzed with BD FACS Aria III (BD Biosciences). For measurement of cellular ROS production, HaCaT cells were treated with diluted LP (1/2, 1/4) for 24 h and then were treated with 10 μM of 5-(and 6)-carboxyl-2′,7′-dichlorodihydro fluorescein diacetate (DCF-DA; Molecular Probes) for 30 min at 37 °C. Intracellular ROS levels was detected using a FACScan flow cytometer (BD Biosciences) with excitation and emission settings at 488 and 530 nm, respectively.

### Mitochondrial membrane potential and Mitochondrial superoxide assay (∆Ψm assay)

The mitochondrial membrane potential (∆Ψm) of intact cells was measured by using JC-1(lipophilic cationic probe 5,5V, 6,6 Vtetrachloro-1,1V 3,3 V-tetra ethylbenzimidazolcarbocyanine iodide, Molecular Probes, Eugene, OR, USA), and Mitochondrial superoxide assay as described previously [79]. The change in ∆Ψm was measured by BD FACSAria III cell sorter (BD Biosciences) and microscope.

### Extracellular ROS and NO production

For extracellular ROS, the supernatant was treated with hydrogen peroxide assay kit (BioVision, Inc, CA, USA) and incubated at room temperature for 30 min. N-cetyl L-cysteine (NAC, Sigma-Aldrich, St Louis, MO, USA) was used for inhibition of ROS generation. The extracellular Nitric oxide production was determined by measuring its stable nitrite, using a Nitric Oxide Assay kit (Abcam, Cambridge, UK) according to the manufacturer’s protocol. The NO inhibitors NG-Methyl-L-arginine acetate salt (500 μM, L-NMMA, Sigma, M7033) and MnTBAP (400 μM, MnTBAP, Calbiochem), were diluted in medium to an indicated concentration. To investigate the efficacy of the NO inhibitors and ROS scavenger, cells were pretreated for 1 h with NO inhibitors prior to a LP treatment. The concentration of H_2_O_2_ and NO in the supernatant was determined using a microplate reader (Epoch 2, BioTek Instruments, Inc, Winooski, VT) with excitation at 540 nm.

### Transmission electron microscopy

Human keratinocyte cells were treated with LP diluted in 1/2, 1/4, and fixed with glutaraldehyde (2%). For transmission election microscopy observation, as previously described [79], all thin sections were observed with an electron microscope (JEOL, Tokyo, Japan, JEM-1011) at an acceleration voltage of 80 kV, and images were analyzed with the Camera-Megaview III Soft imaging system.

### In vivo study with dorsal full-thickness skin wound murine model

Animal care and procedures were in accordance with the National Institutes of Health Guidelines for the Care and Use of Laboratory Animals, and all experiments were approved by the Committee for Ethics in Animal Experiments of the Ajou University School of Medicine (approval number; 2021-0012). We conducted an experiment with twenty 6-week-old female Sprague Dawley rats (10 control animals, 10 LP-treated animals were randomly allocated with no blinding) with body weight in the range of 150–200 g, which is a generally employed population that can confirm the statistical significance of the study results in the field. The animals were anesthetized with isoflurane (2%, Forane, Abbott Laboratories, Abbott Park, IL), and two full-thickness round wounds were created using a biopsy punch of 8 mm diameter (IntegraLifeSciences, Plainsboro, NJ, USA) on both side of their upper back midline, after shaving the hair on their backs. LP-soaked gauze was applied to the wound for LP treatment for 15 min every two days from the day the wound was created, and PBS-soaked gauze was applied to the control wound at the same time. For 14 days from the date of formation of the wound, the wound was recorded with a ruler by photograph. The wound area was taken and randomly calculated with no blinding. The surface wound area was quantified using ImageJ software (NIH, Bethesda, MD). The relative wound area of initially created wound was calculated as (wound area of the date of measurement/area of original wound on day 1) × 100. For the calculation of relative wound area for wound healing analysis, an average of 7 animals in each group was used. The animals were euthanized on days 7 and 14 from the day of wound creation, and skin samples including the wound region were harvested for further histological assessments.

### Histological analysis by hematoxylin and eosin staining

The skin samples were fixed in 4% paraformaldehyde (in 0.1 M PBS, pH 7.4) and mounted into paraffin. After dehydration and embedding in paraffin, the blocks were serially cut from midline sections (5 μm thickness) using a microtome (Leica RM2125 RTS (ka2.225), Germany) and were rehydrated with distilled water and stained with hematoxylin and eosin for histological analysis. Images of stained slides were taken to analysis using an EVOS2 optical microscope (Thermo Fisher Scientific, Waltham, MA, USA) at 100× magnification.

### Immunohistochemistry study

Immunohistochemistry was performed on paraffin-embedded skin wound tissue sections prepared on glass slides. Briefly, specimens were incubated in blocking solution with anti-Ki-67 (1:200), anti-NOX3 (1:200), anti-integrin β1 (1:100), anti-p-FAK (1:150), and anti-p-paxillin (1:200) overnight at 4 °C. Immunolabeling was performed after 3 washes in PBS and stained with Liquid DAB + Substrate Kit (GBI Labs). DAB-stained tissue slides were captured by EVOS2 microscopy. The images were used for quantification of stained area by the ImageJ software program (NIH, Bethesda, MD, USA).

### Statistical analysis

The data was analyzed by a one‐way analysis of variance (ANOVA). Following this ANOVA, we performed a post hoc Tukey’s test using SPSS ver. 20.0 (IBM Corp., Armonk, NY). The *p*-value lower than 0.05 (*p* < 0.05, *p* < 0.01, *p* < 0.001) were considered statistically significant.

## Supplementary information

Supplementary information

Full and uncropped western blots

reproducibility checklist

Supplementary video

## Data Availability

All data generated or analyzed during this study are included in this published article and its supplementary information files.
