# Supplementary material for: Liquid plasma as a treatment for cutaneous wound healing through regulation of redox metabolism
Source: Cell Death Dis. 2023 Feb 13;14(2):119. doi: 10.1038/s41419-023-05610-9 (PMC9925775; doi:10.1038/s41419-023-05610-9)
Supplement: Supplementary file 1 — Supplementary information [file 41419_2023_5610_MOESM1_ESM.docx]

Supplementary Information

Liquid Plasma as a Treatment for Cutaneous Wound Healing Through Regulation of Redox Metabolism

*Hye Ran Lee^1,2†^, Sung Un Kang^3†^, Haeng Jun Kim^3^, Eun Jong Ji^3^, Ju Hyun Yun^3^, Sungryeal Kim^4^, Jeon Yeob Jang^3^, Yoo Seob Shin^3^, Chul-Ho Kim^3*^
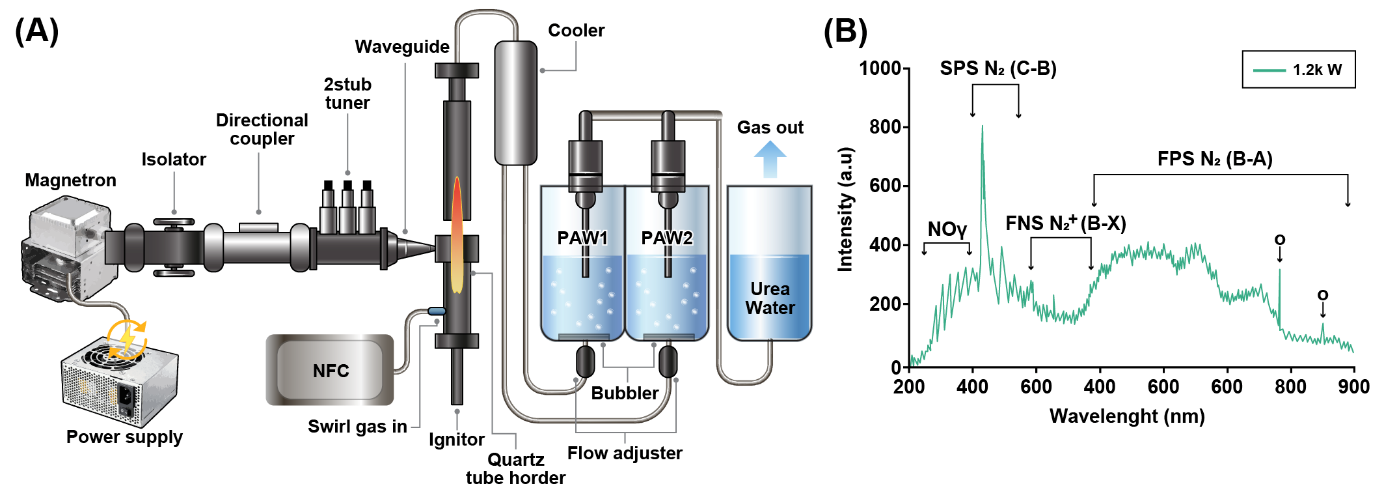
*

**Supplementary Fig. 1.** Liquid plasma (LP) generating system used in this study. (A) Photograph of our plasma-generating system. In order to generate the LP, 20-LPM (liters per minute) air as a swirl gas enters the quartz tube through the quartz tube holder. The heated nitric oxide gases generated in the plasma torch flame enters a cooler to lower the gas temperatures close to room temperature. The cooled gases are introduced into two plasma activated water (PAW) basins via porous bubblers to increase the dissolution rate of nitric oxide gases. The urea water tank was installed at the rear end to remove the nitric oxide and nitric dioxide gas remaining for decrease air pollution. (B) Optical emission spectra of the gas mixture plasma according to the electric intensity (2.45 GHz, 1.2 kW) in the range of 200–900 nm.

**Supplementary table 1.** Antibodies Information.

| **Name** | **Cat. No** | **Dilution ( Method )** | **Company** |
| --- | --- | --- | --- |
| Integrin b1 | #34971 | 1:1000 ( WB ), 1:50 ( IHC ) | Cell signaling |
| Integrin b3 | #13166 | 1:1000 ( WB ) | Cell signaling |
| Integrin b5 | #4708 | 1:1000 ( WB ) | Cell signaling |
| p-FAK(tyr397) | #8556 | 1:1000 ( WB ) | Cell signaling |
| p-FAK (tyr397) | 700255 | 1:50 ( IHC ) | Invitrogen |
| FAK | #3285 | 1:1000 ( WB ) | Cell signaling |
| p-paxillin (tyr118) | #2541 | 1:1000 ( WB ) | Cell signaling |
| p-paxillin (tyr118) | 44-722G | 1:50 ( IHC ) | Invitrogen |
| NOX3 | ab81864 | 1:1000 ( WB ) | Abcam |
| NOX3 | NBP2-41292 | 1:50 ( IHC ) | Novus biologicals |
| PCNA | #13110 | 1:1000 ( WB ) | Cell signaling |
| Ki67 | MA5-14520 | 1:50 ( IHC ) | Invitrogen |
| GAPDH | #13166 | 1:1000 ( WB ) | Cell signaling |
| Secondary antibodies | #7074, 7076 | 1:3000 ( WB ) | Cell signaling |
| Secondary antibodies | #31460, #31430 | 1:250 ( IHC ) | Invitrogen |

Abbreviations: WB- Western blot; IHC-Immunohistochemistry,

**
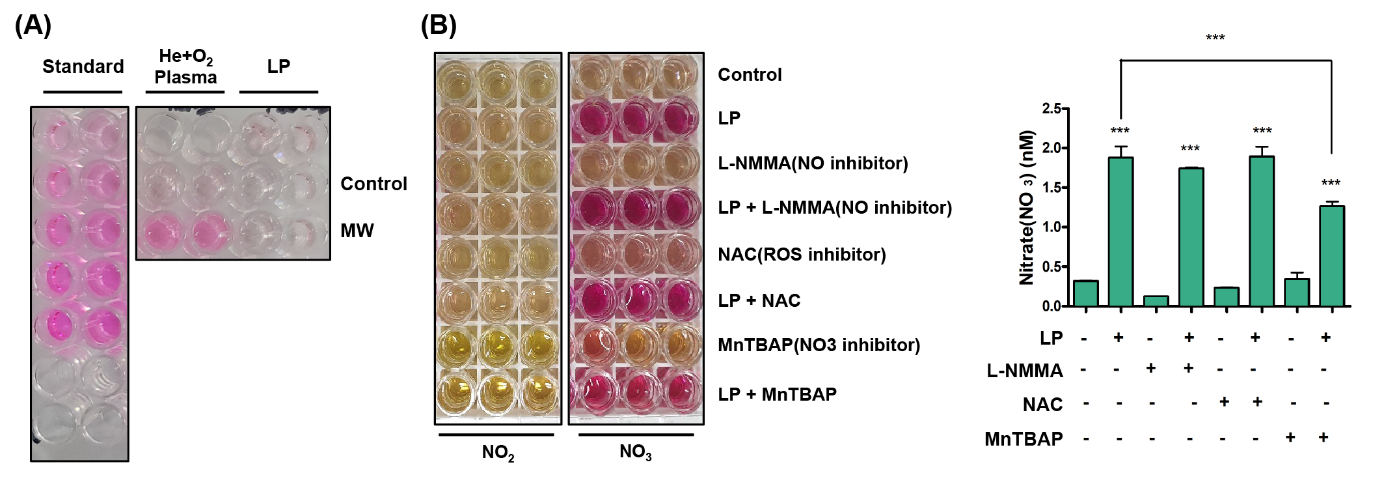
**

**Supplementary Fig. 2.** Analysis of LP on extracellular RNS/ROS generation. (A) H_2_O_2_ release measured via Amplex Red assay. LP does not increase extracellular ROS. He ^+^ O_2_ plasma used as plasma source. (B) The nitrite anion (NO_2_^-^) and nitrate anion (NO_3_^-^) detected by the NO_3_ detection kit in LP. The levels of extracellular NO_3_^-^, an RNS, increased in the presence of LP. All samples were treated with a positive control with either no inhibitor or one of the signaling inhibitors listed: Inhibitors #1 (L-NMMA, a nitric oxide synthase inhibitor), #2 (NAC, ROS scavenger), and #3 (MnTBAP, peroxynitrite scavenger). Among them, only #3 treatment resulted in a significant decrease in mean extracellular nitric oxide concentration when added to LP (***P <0.005, and *P <0.05). Bar graphs represent means ± SD of three independent experiments. ***P <0.001.

**
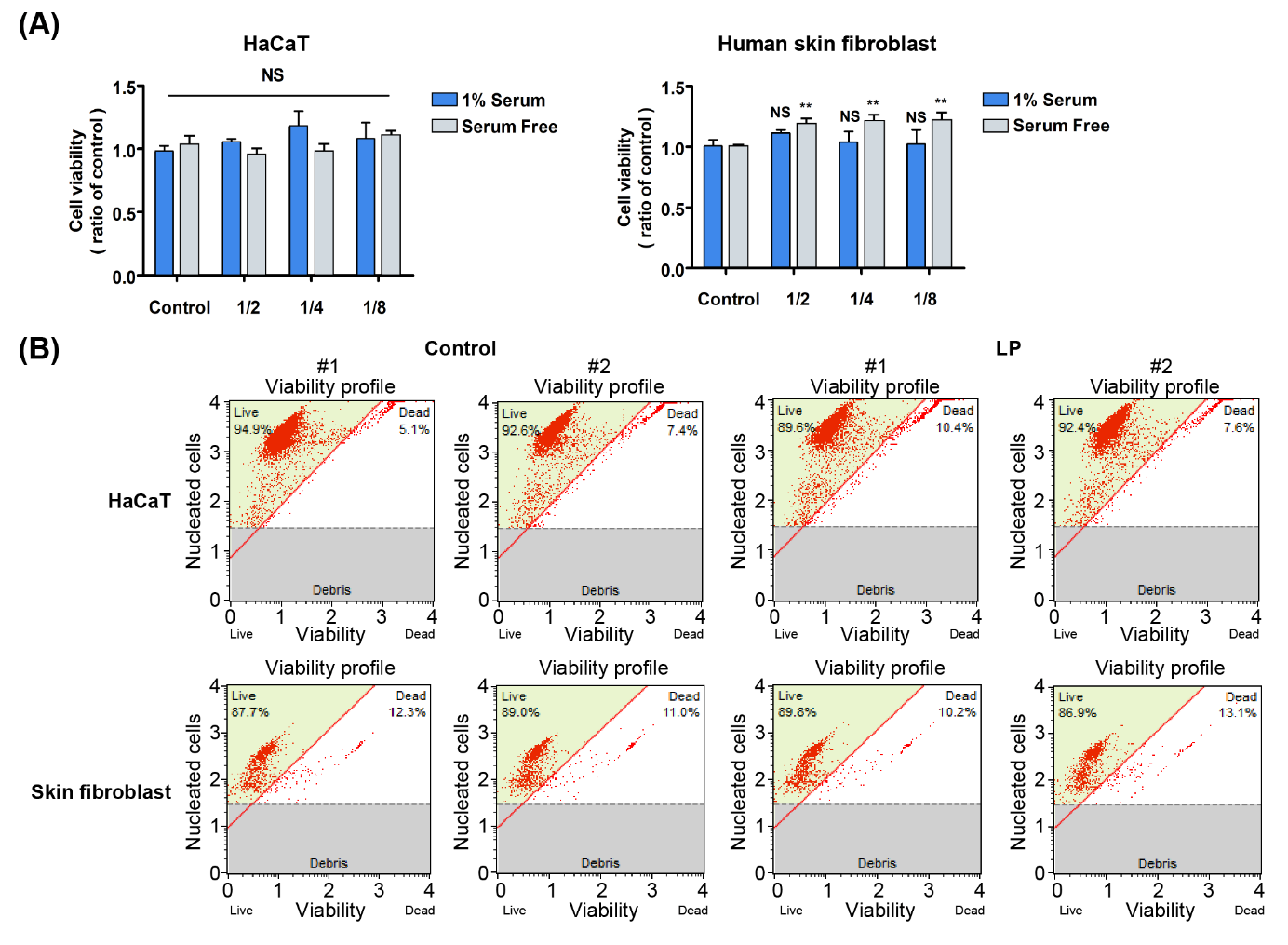
**

**Supplementary Fig. 3.** LP does not induce cytotoxicity in the human keratinocyte cell lines (HaCaT) and human skin fibroblasts. (A) Cell viability evaluated by the MTT assay. (B) Analysis of cell viability using the Muse ® Cell Analyzer. The percentage of live cells detected in the presence of the indicated treatment is shown (N = 6). Asterisks indicate statistically significant differences (*P <0.05, **P <0.01, ***P <0.001).

**(A) Primary Epidermal Keratinocytes**

**LP ( 1/4 )**

**Control**

*^
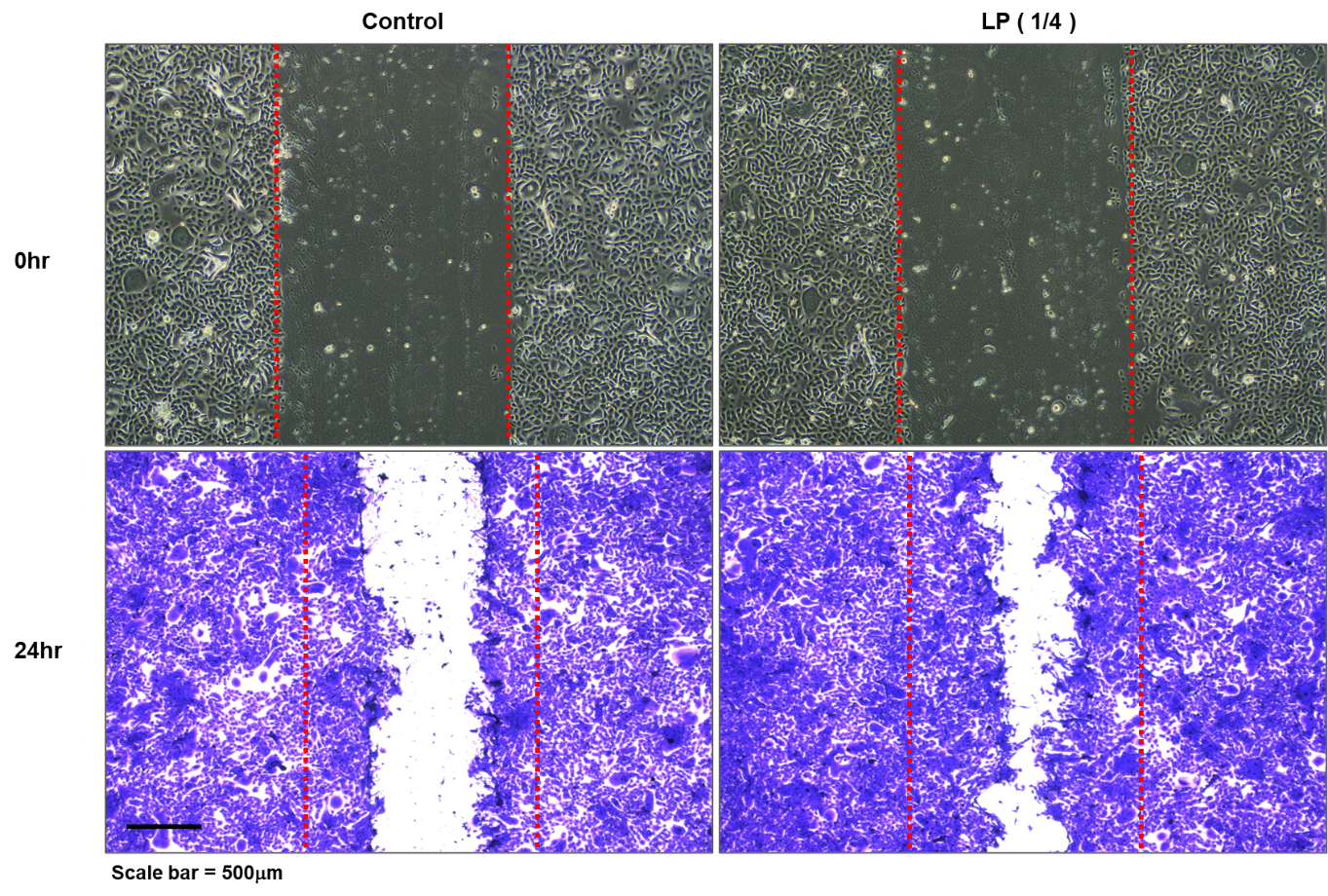
^*

*^
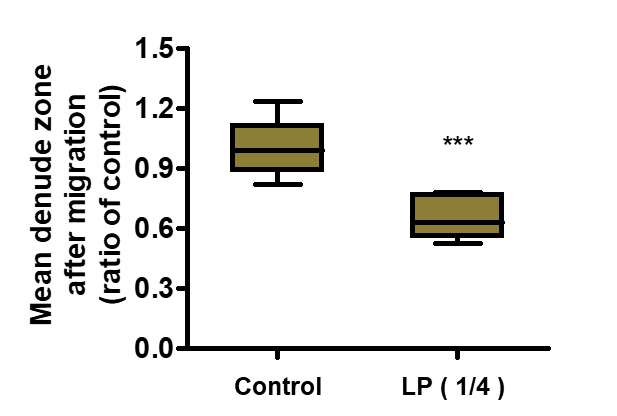
^*

**(B) Primary Dermal Fibroblast**

**LP ( 1/4 )**

**Control**

*^
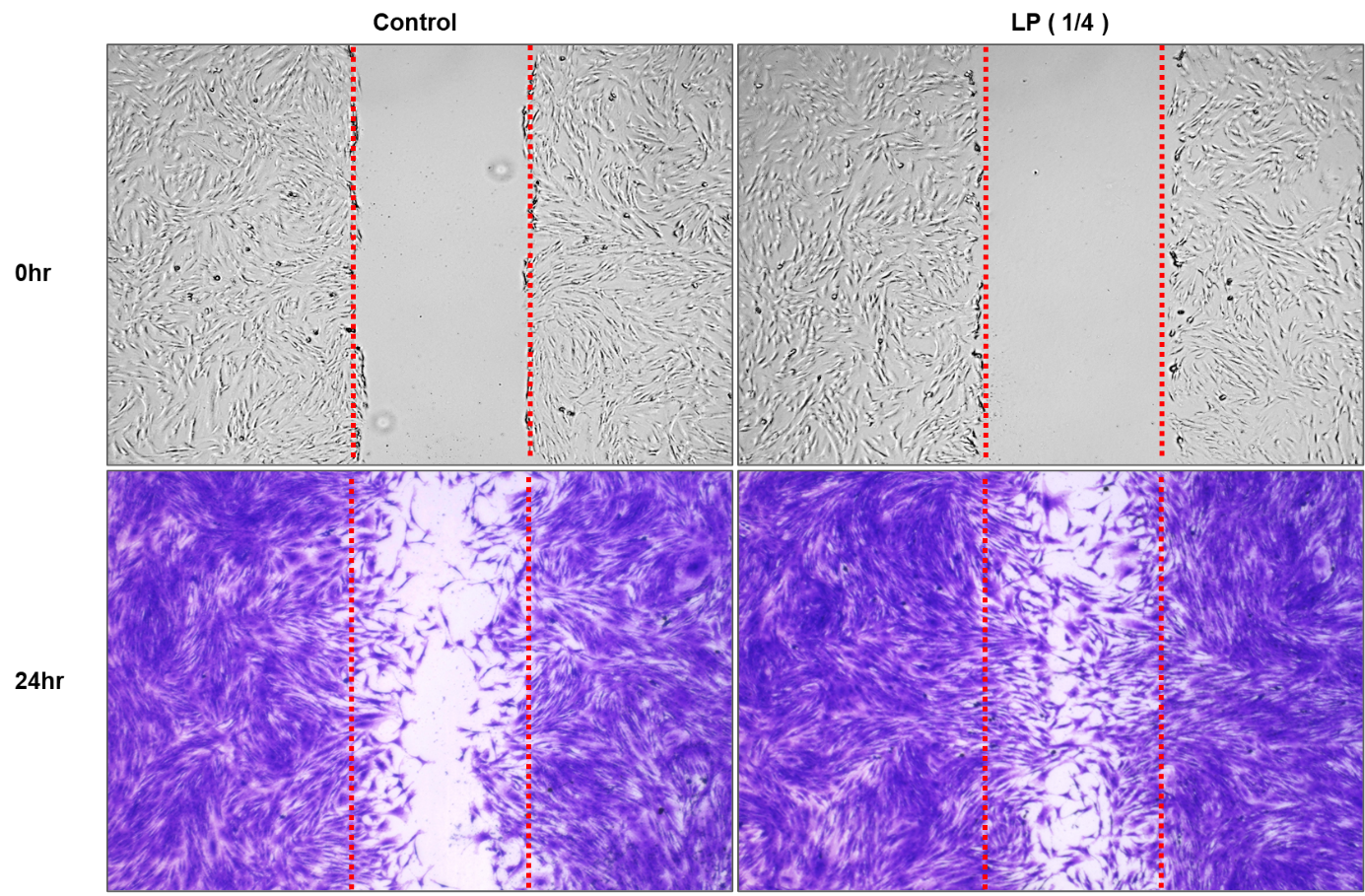
^*

**Supplementary Fig. 4. Effects of LP treatment on cell migration.**

Scratch wound migration assay after LP treatment on human primary keratinocyte (A), and fibroblast (B) cells. The cells were plated in six-well plates, grown to 90% confluency, and with a monolayer denuded with a sterile pipette tip. Scratch wound migrations (scale bars = 500 μm) were documented by photography after 24 h of incubation. The data graph presents the mean ±

standard deviation of three independent experiments. ***P <.001.


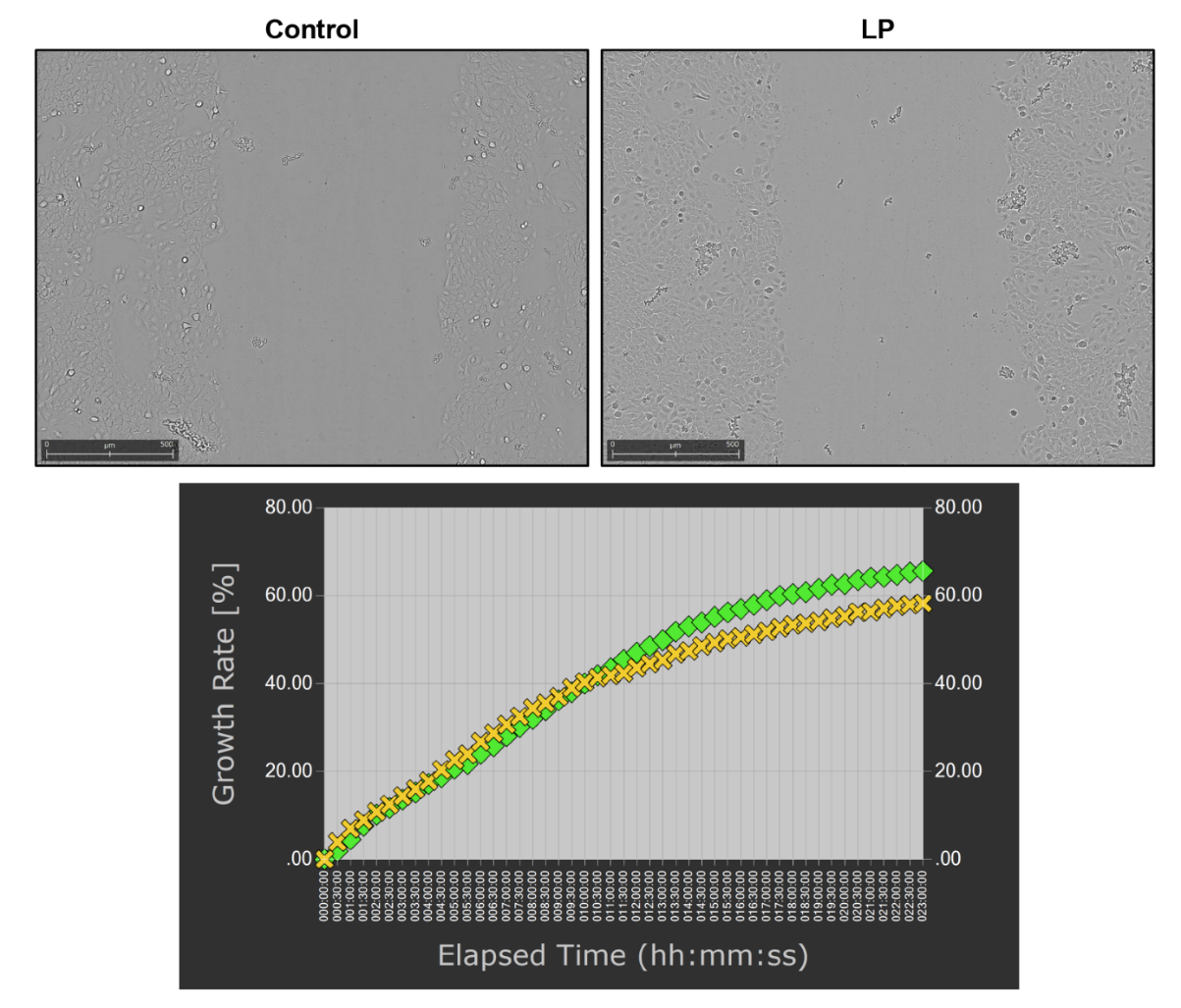


**Supplementary Fig. 5.** Wound healing assay by live cell imaging on the JuLi Br Live Cell Analyzer. Relative density of HaCaT cells in created scratch wound at different time points in a 23-h period, demonstrated by graph as cellular growth rate (%). The measurements are from wounds made on a monolayer of HaCaT cells cultured in the presence of different coating treatments: control (DI water 1/2) and LP (1/2). At 10 h after the start of cell migration, the growth rate of LP-treated HaCaT cells was faster than the control. Live video of each group is presented as an additional supplementary video. Graph data represent the mean ± SD of three independent experiments. *p <0.05. Yellow bar: growth rate of the control group; Light green bar: growth rate of LP-treated group.

**Description of Supplementary Video**

Description: Live video of migration of HaCaT cells in the control group and LP-treated group.


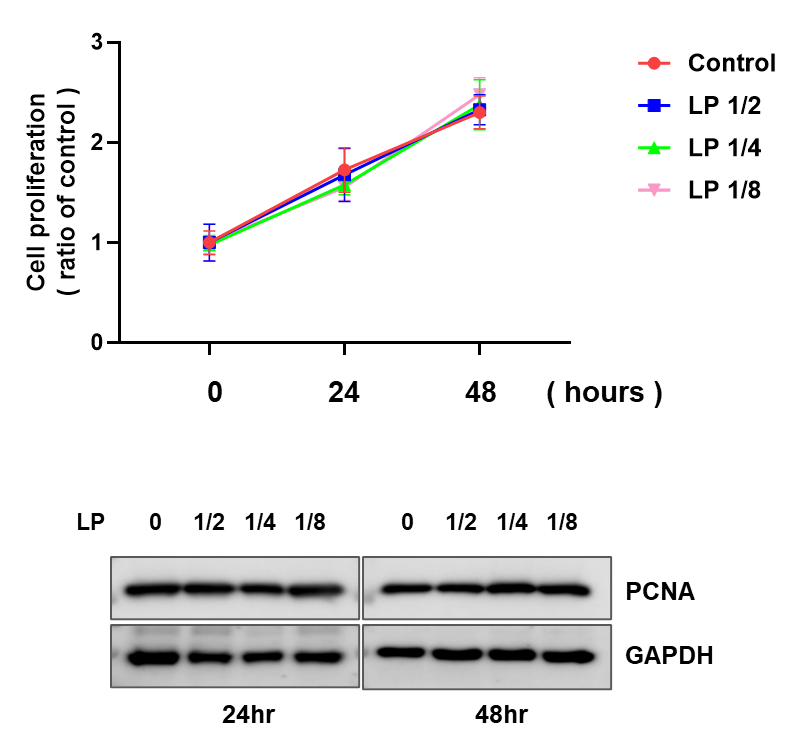


**Supplementary Fig. 6.** Evaluation of cell proliferation on LP treatment in the human keratinocyte cell lines (HaCaT) and the quantitative results of the cell density showed that treatment with LP did not induce cell proliferation using BrdU assay (upper panel). Western blot analysis on PCNA (a proliferating cell nuclear antigen) in control and LP treated groups did not show a significant difference in expressions (down panel).
