# Supplementary material for: Liquid plasma as a treatment for cutaneous wound healing through regulation of redox metabolism
Source: Cell Death Dis. 2023 Feb 13;14(2):119. doi: 10.1038/s41419-023-05610-9 (PMC9925775; doi:10.1038/s41419-023-05610-9)

Source Data_Figure 2A

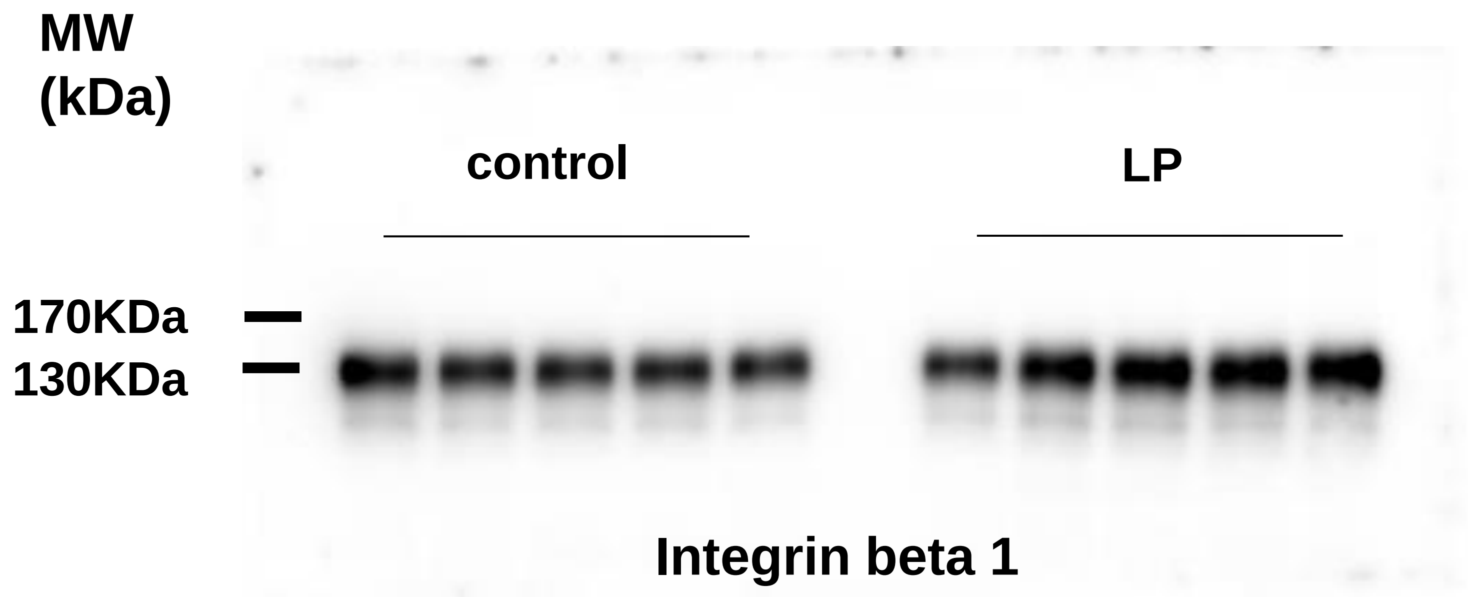

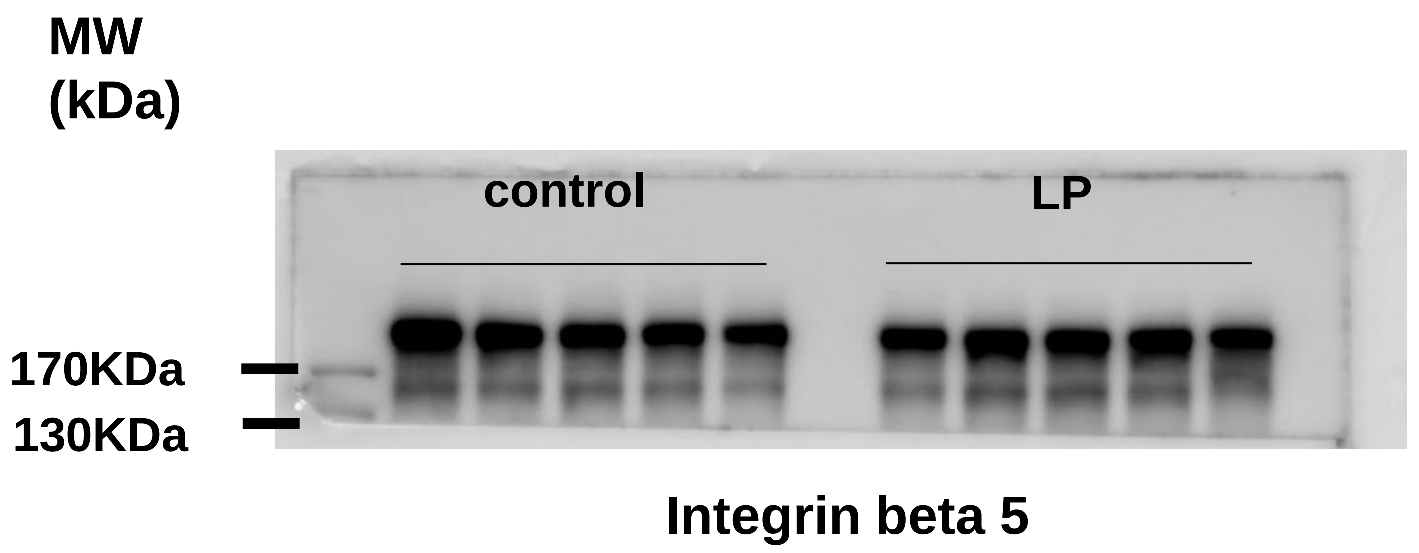

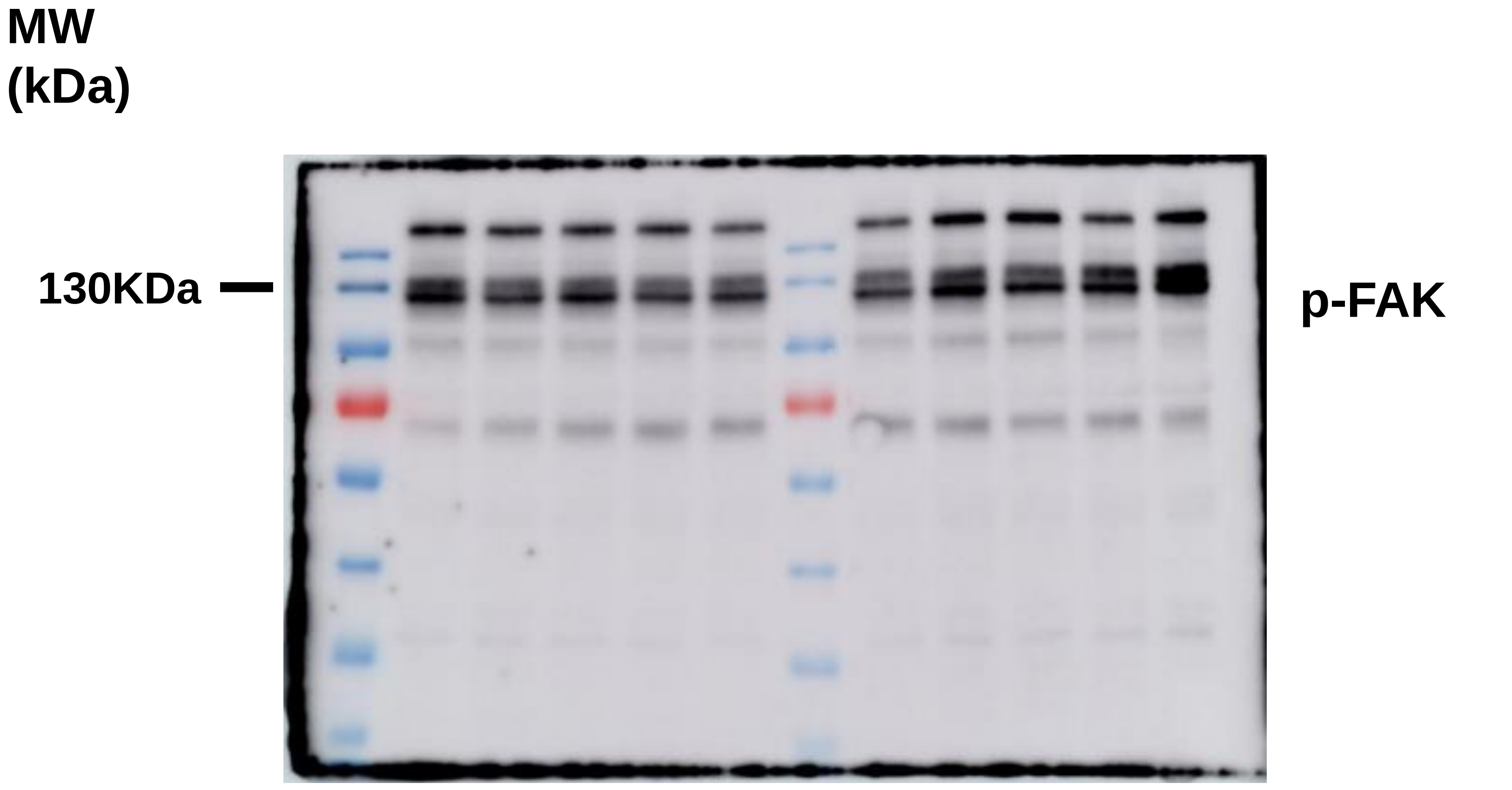

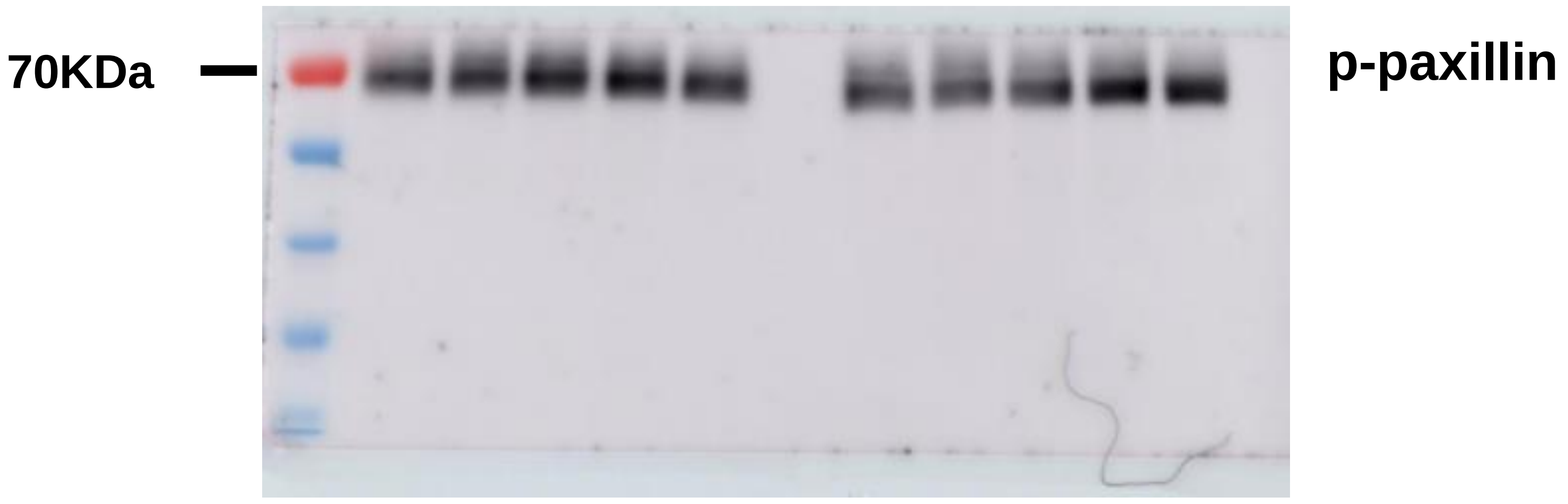

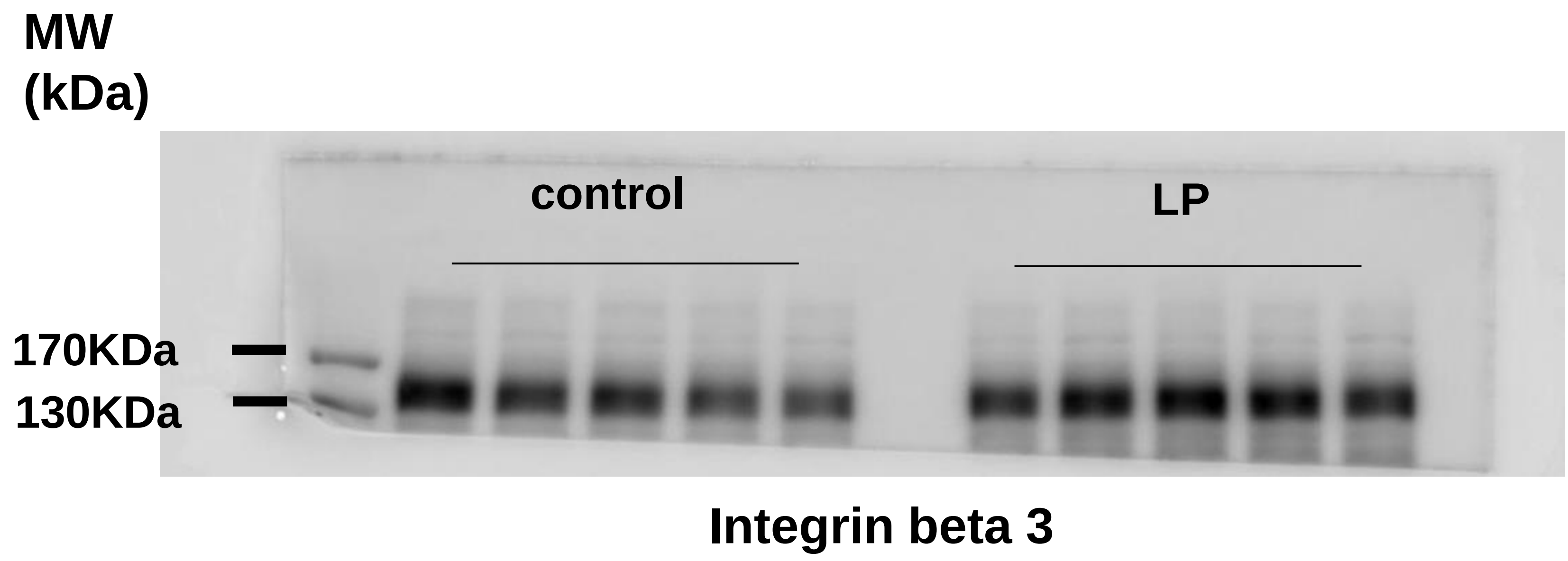

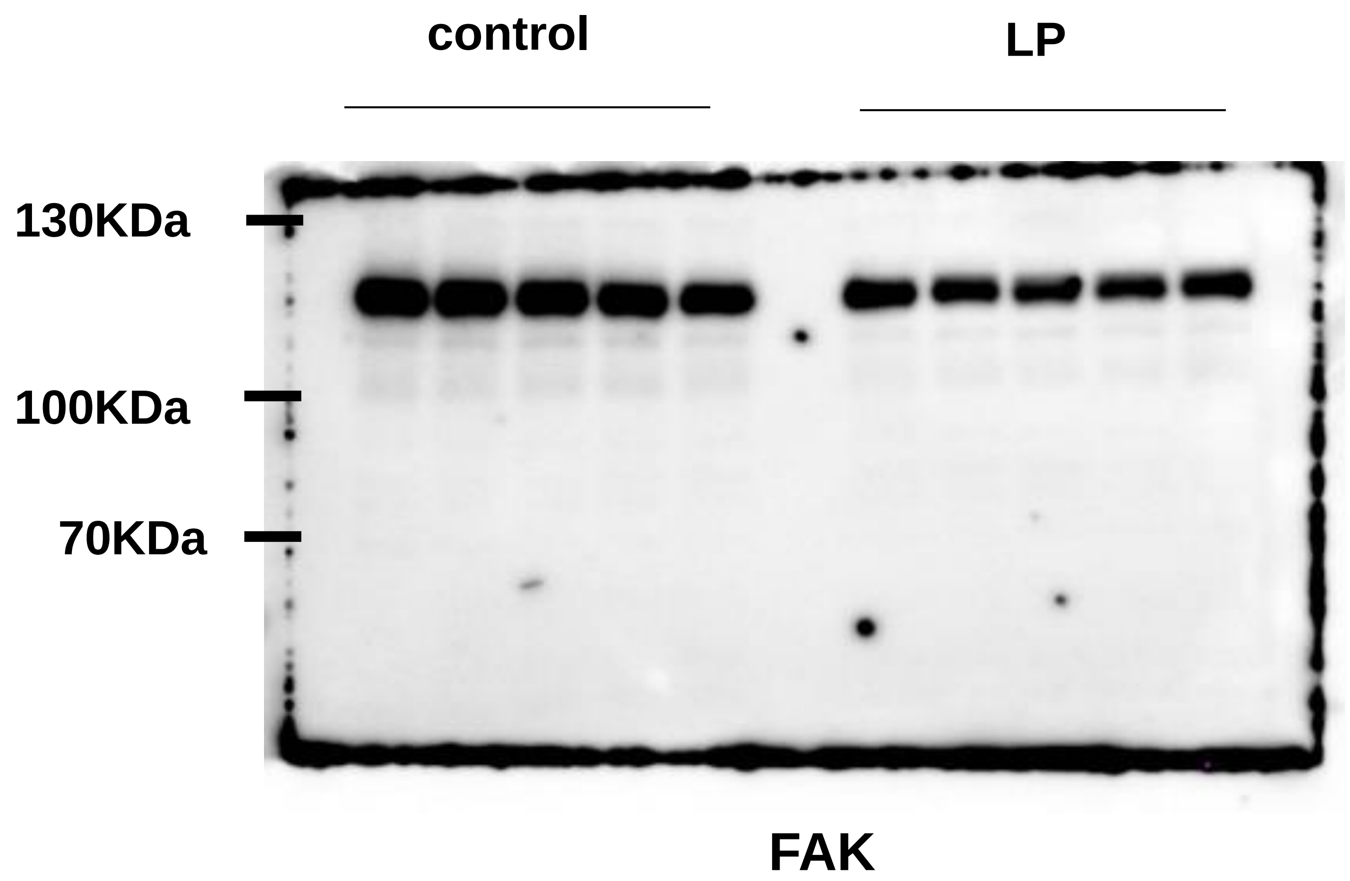

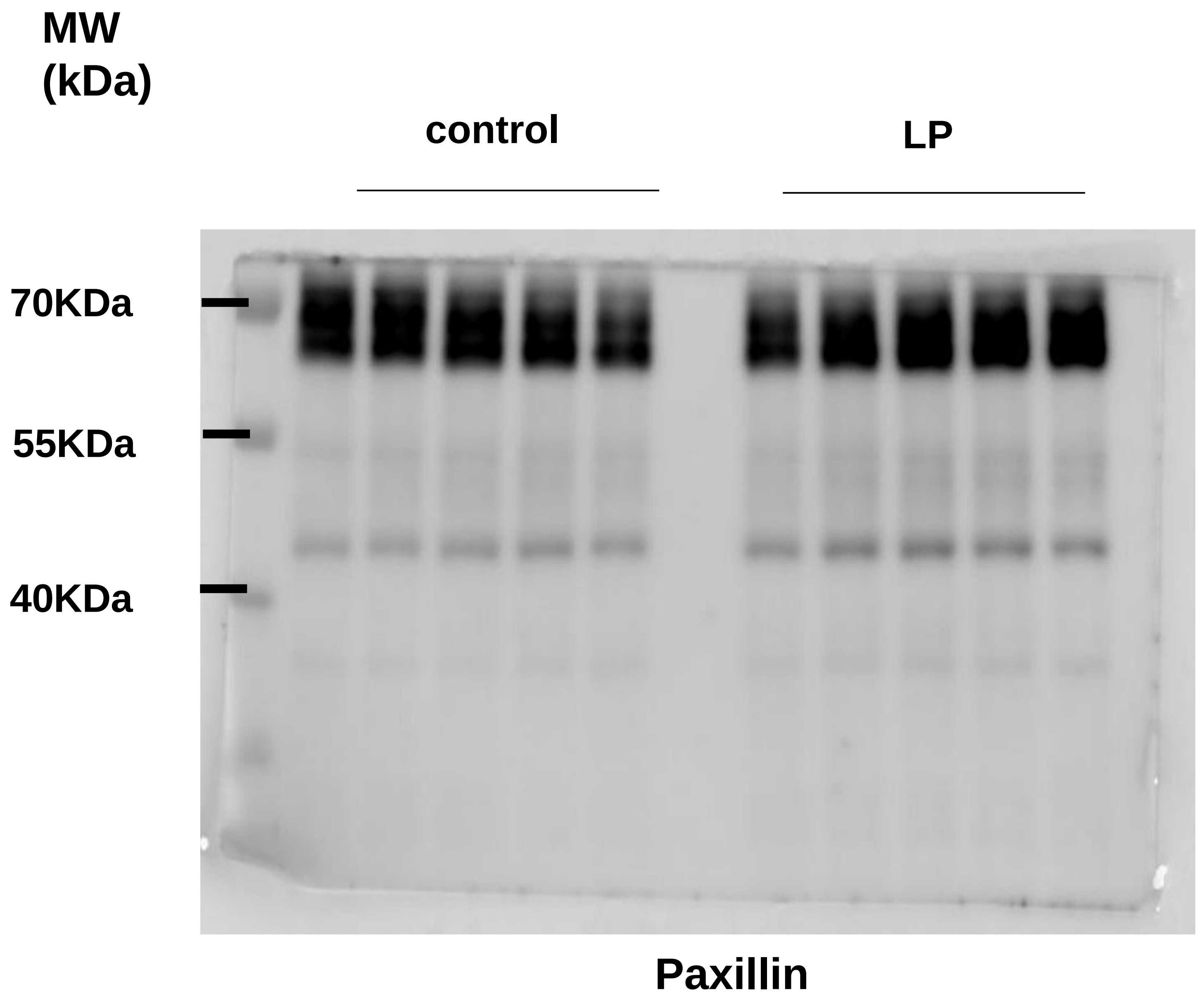

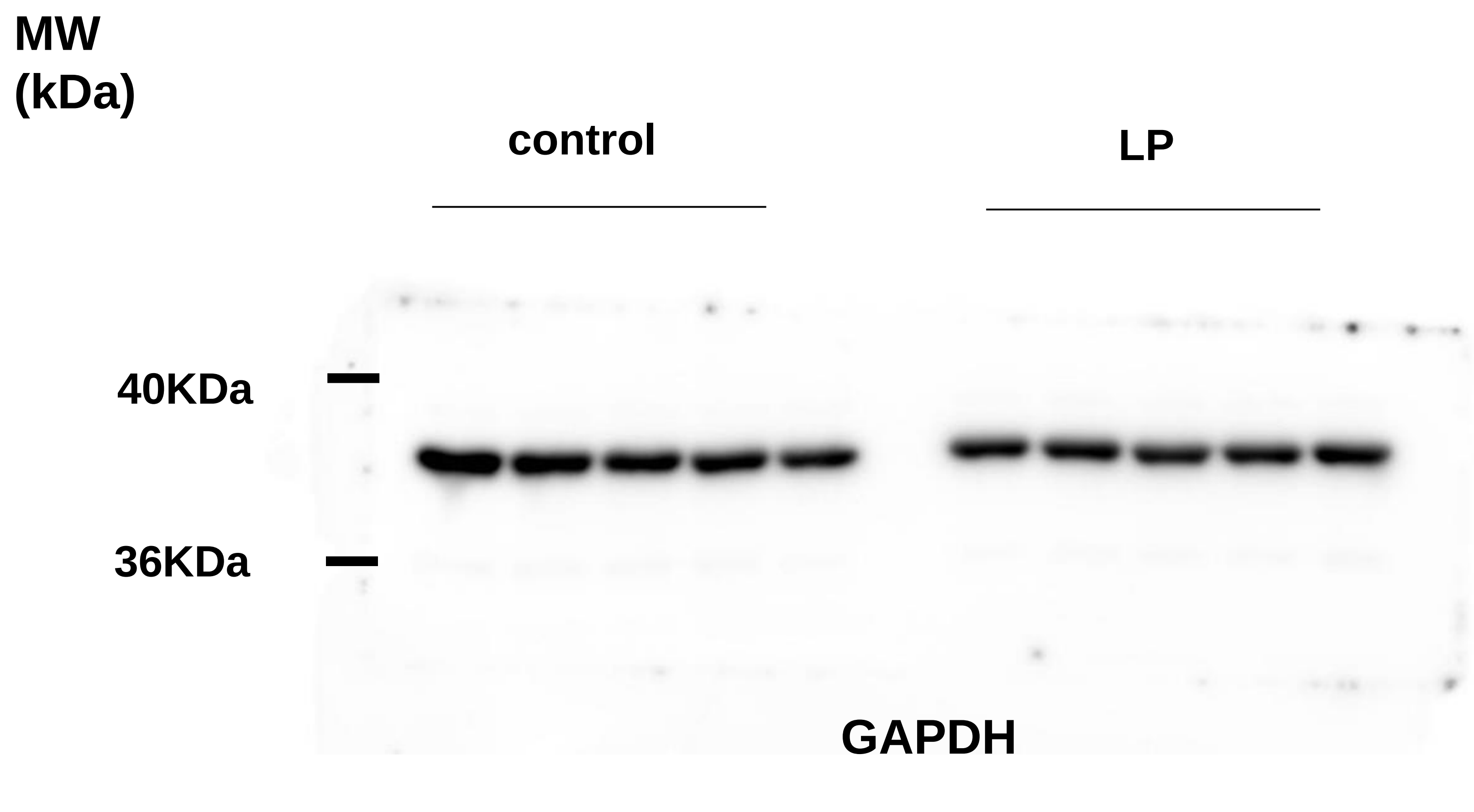

D.

Source Data_Figure 3D

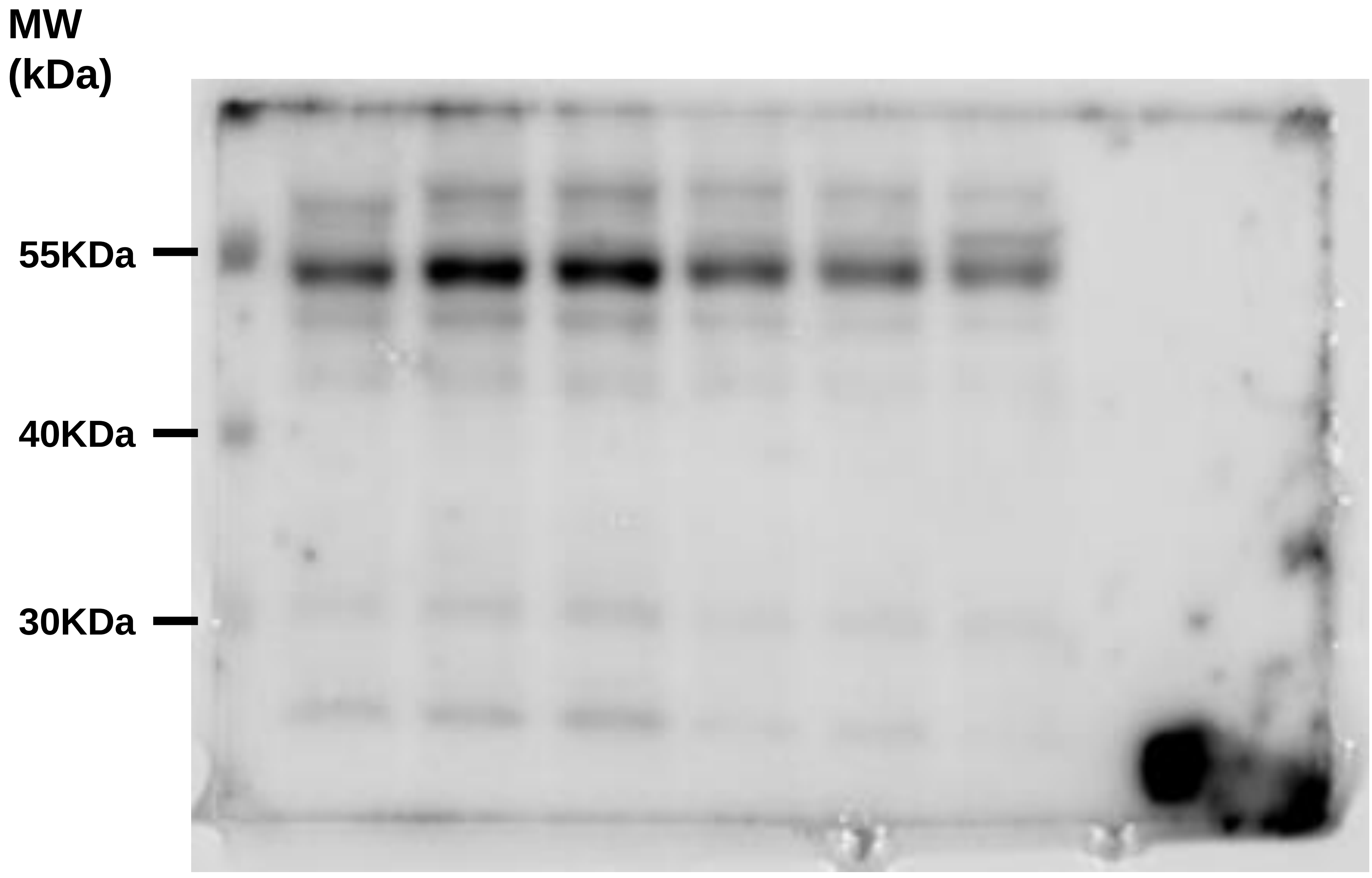

NOX3

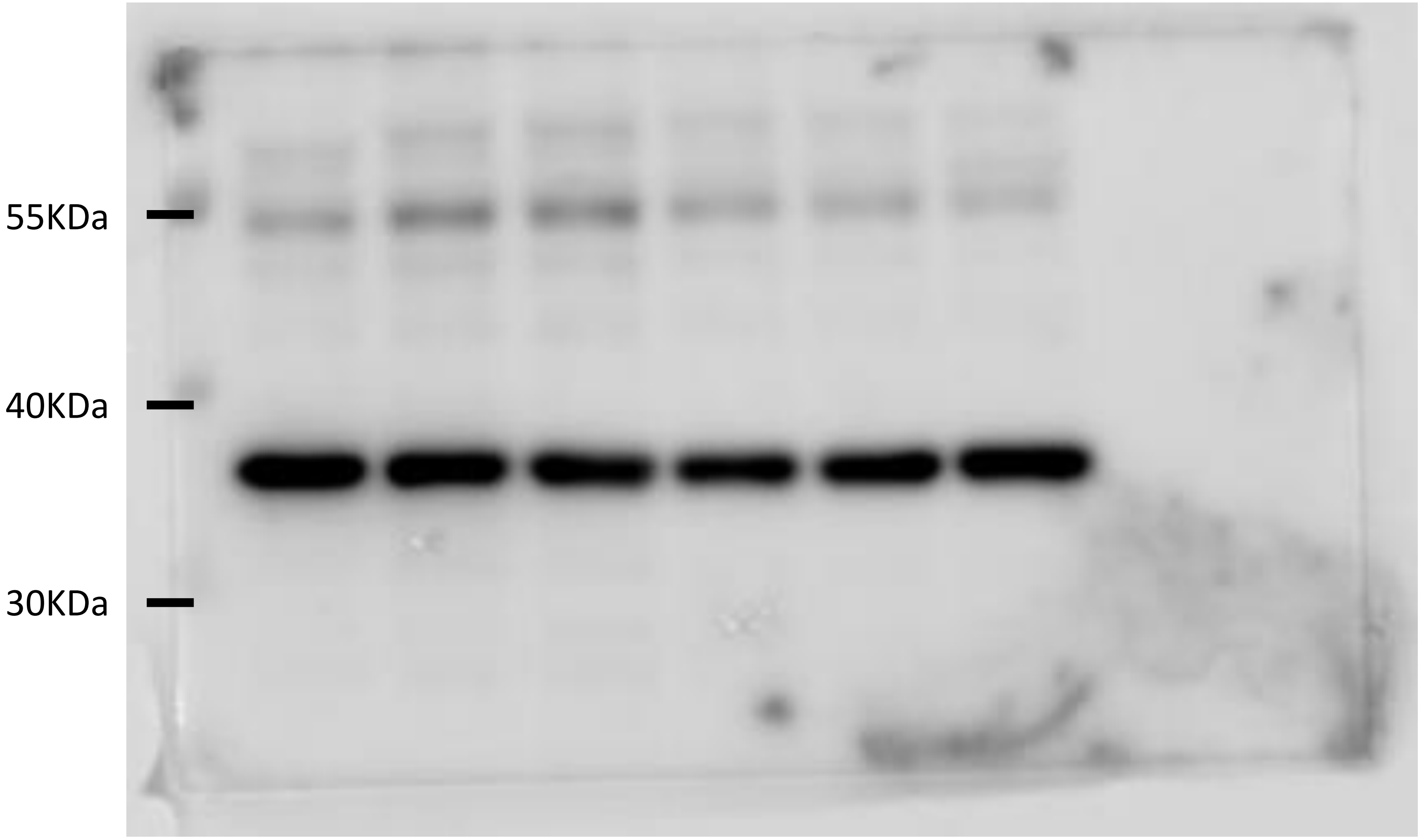

GAPDH

Source Data_Figure 4E

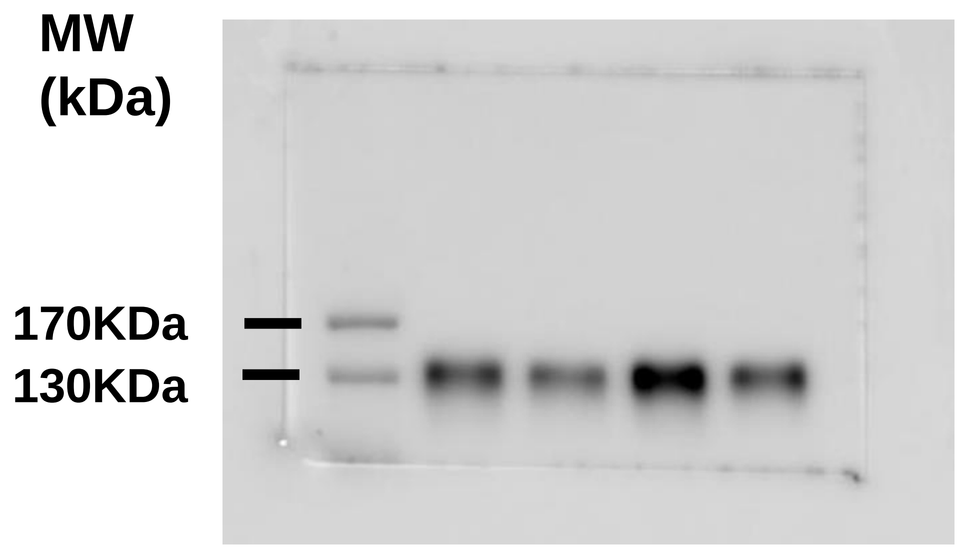

Integrin beta 1

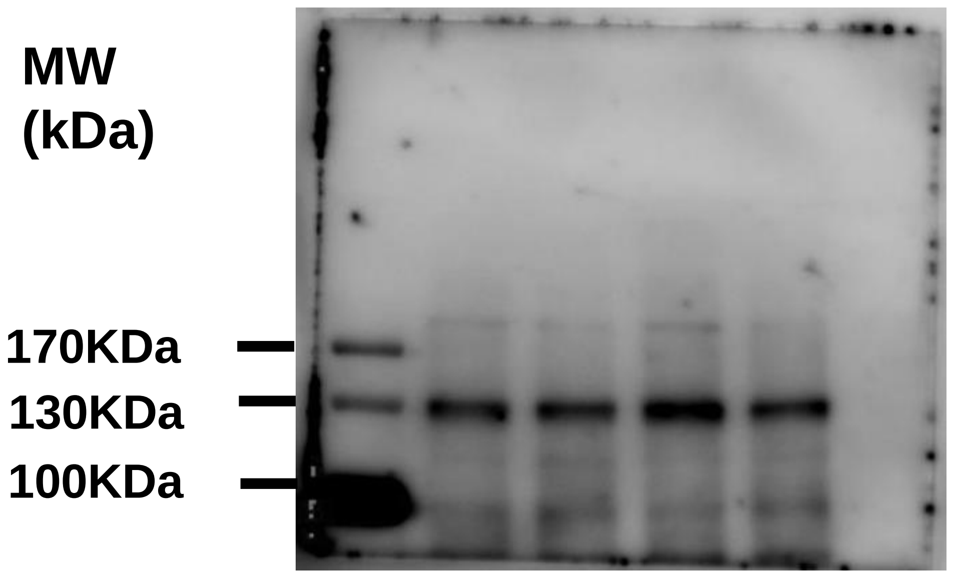

Integrin beta 3

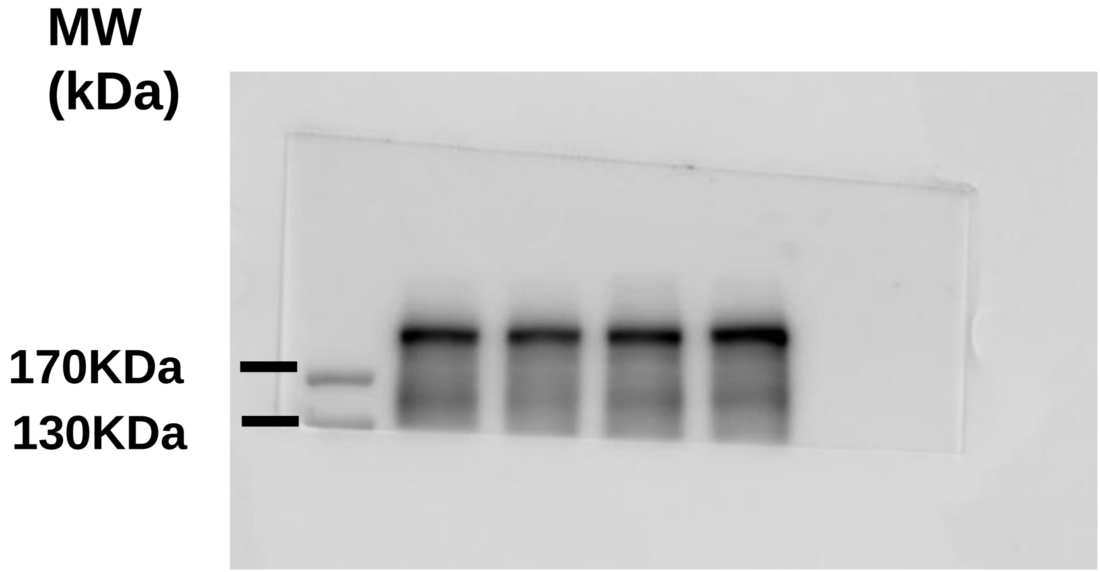

Integrin beta 5

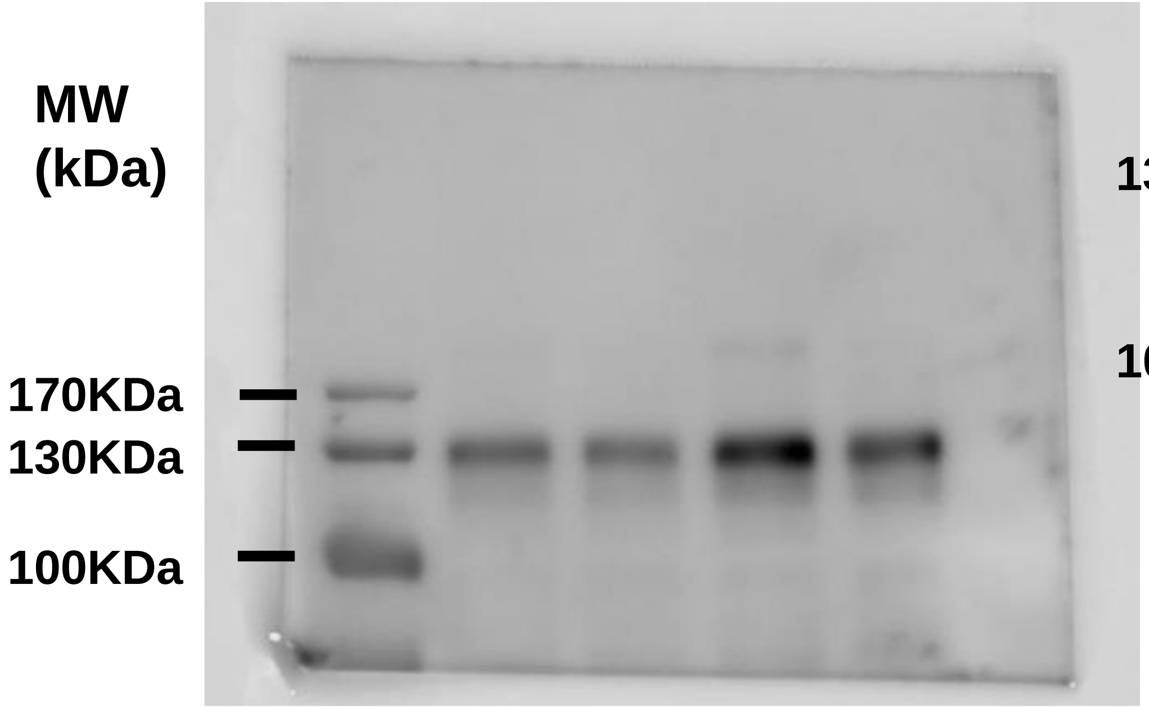

p-FAK (y397)

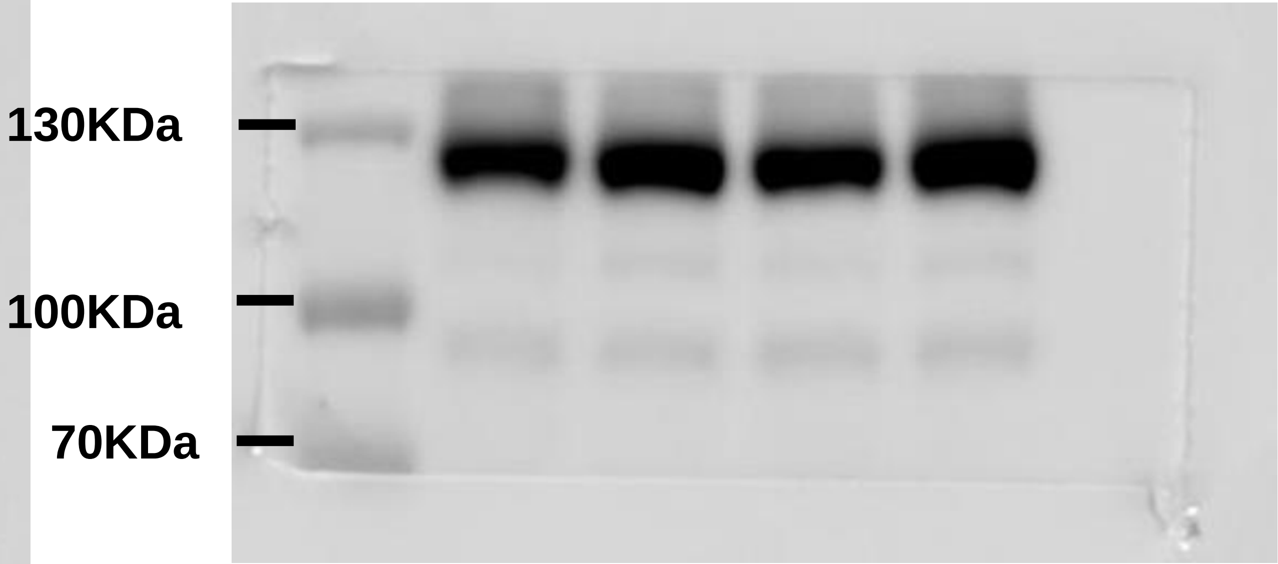

FAK (total)

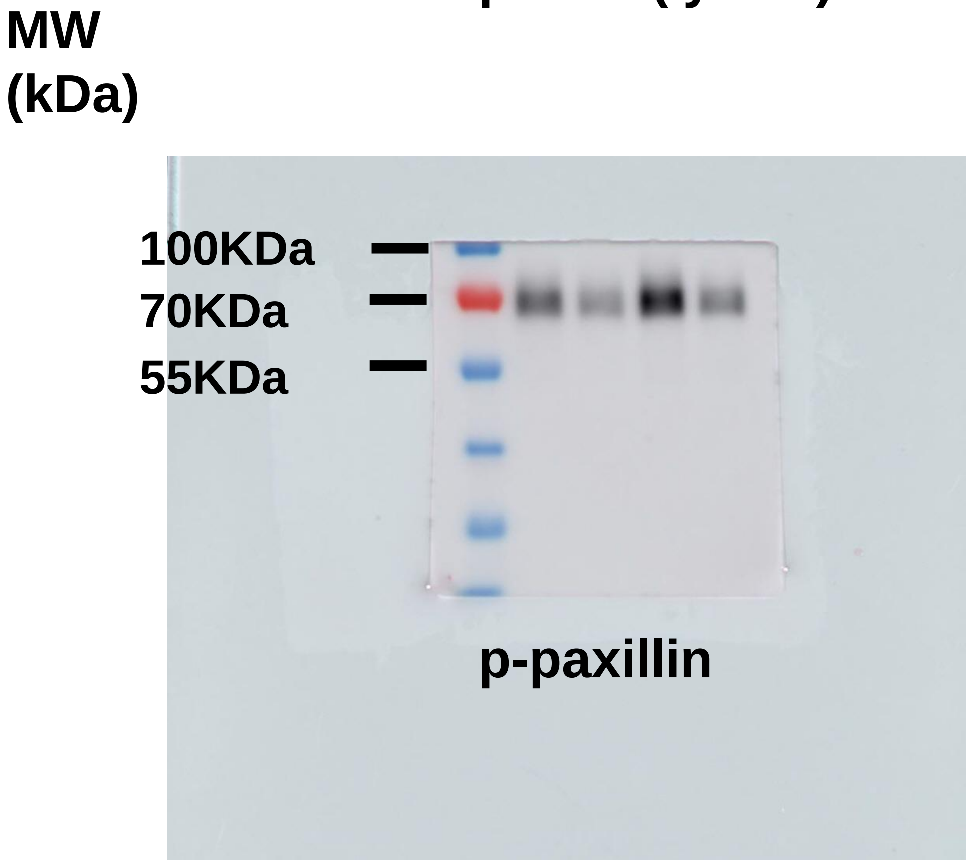

p-paxillin

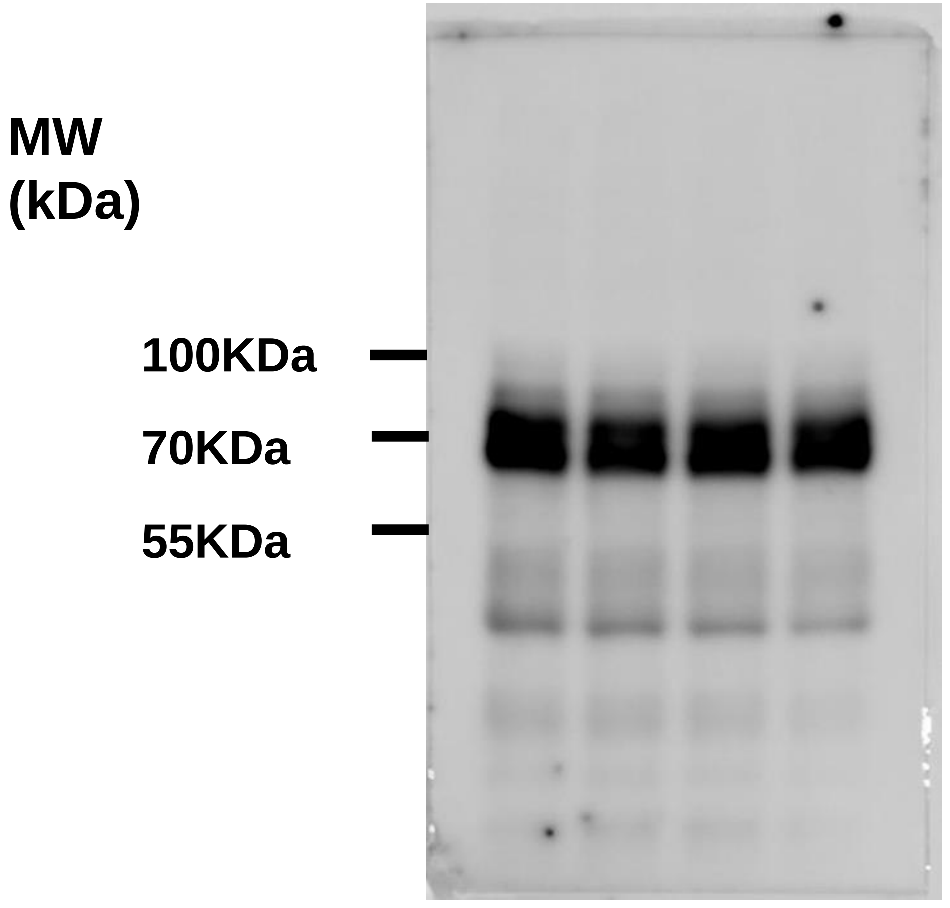

paxillin

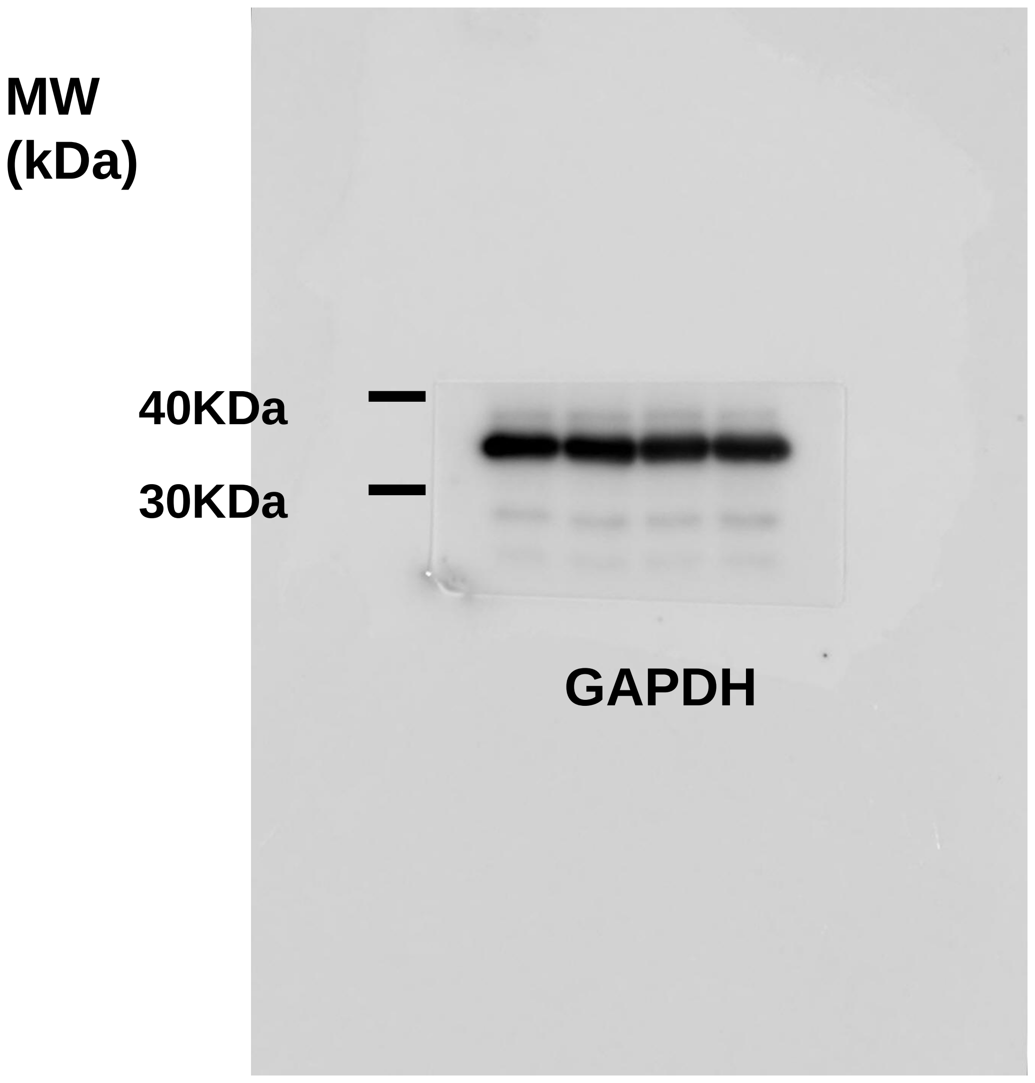

GAPDH

Source Data_Supplementary figure 6

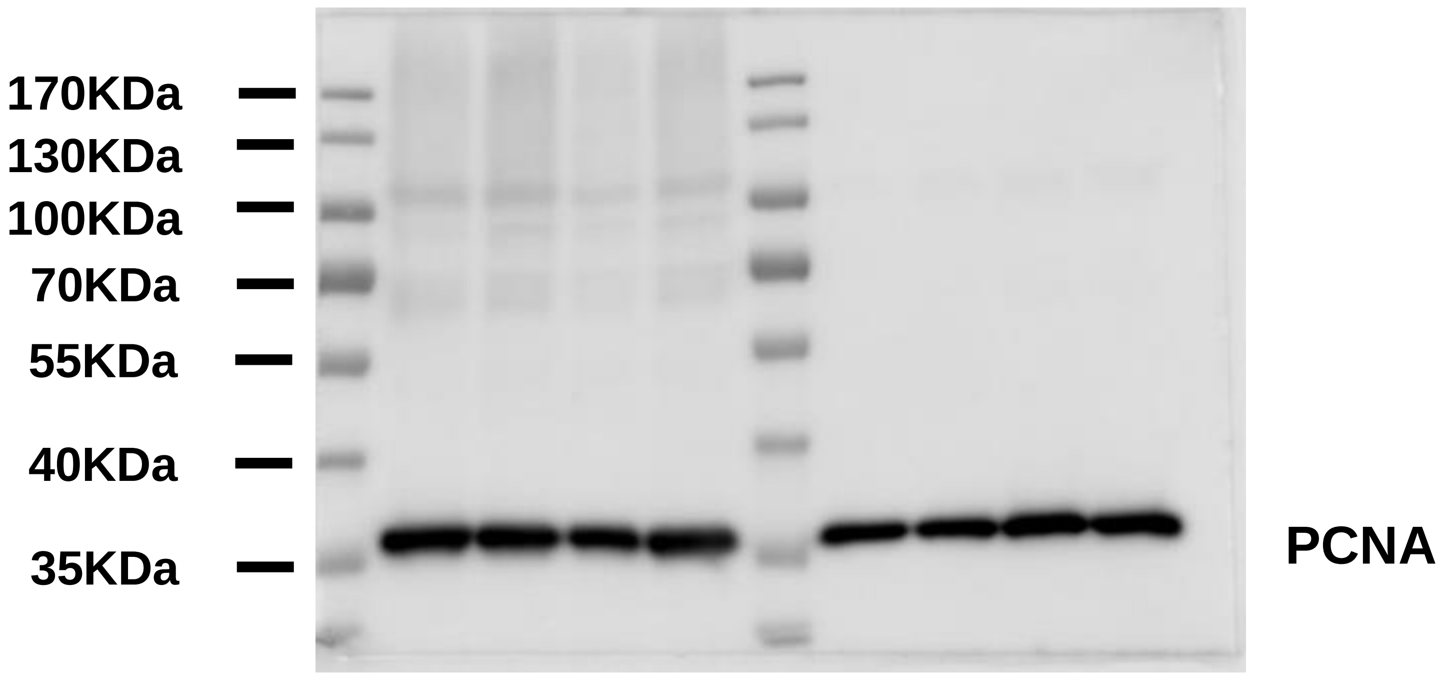

Proliferation marker

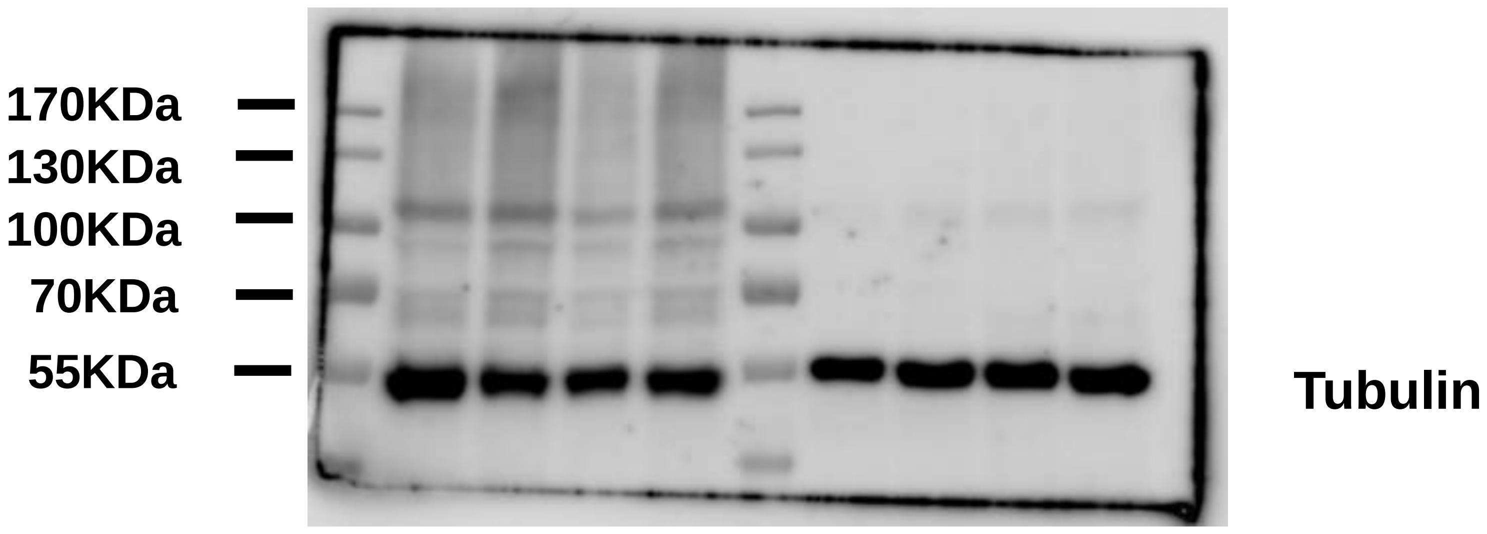

Supplement: Supplementary file 2 — Full and uncropped western blots [file 41419_2023_5610_MOESM2_ESM.pdf]
